# The Non-Coding RNA Landscape of Plasma Cell Dyscrasias

**DOI:** 10.3390/cancers12020320

**Published:** 2020-01-30

**Authors:** Eugenio Morelli, Annamaria Gullà, Roberta Rocca, Cinzia Federico, Lavinia Raimondi, Stefano Malvestiti, Valter Agosti, Marco Rossi, Giosuè Costa, Gianluca Giavaresi, Abdel Kareem Azab, Antonia Cagnetta, Michele Cea, Pierosandro Tagliaferri, Antonino Neri, Nikhil C. Munshi, Giuseppe Viglietto, Pierfrancesco Tassone, Nicola Amodio

**Affiliations:** 1 Dana Farber Cancer Institute, Harvard Medical School, Boston, MA 02215, USA; eugenio_morelli@dfci.harvard.edu (E.M.); annamaria_gulla@dfci.harvard.edu (A.G.); malvestitistefano@gmail.com (S.M.); nikhil_munshi@dfci.harvard.edu (N.C.M.); 2 Department of Experimental and Clinical Medicine, *Magna Graecia* University of Catanzaro, 88100 Catanzaro, Italy; rocca@unicz.it (R.R.); agosti@unicz.it (V.A.); rossim@unicz.it (M.R.); tagliaferri@unicz.it (P.T.); viglietto@unicz.it (G.V.); tassone@unicz.it (P.T.); 3 Net4Science srl, *Magna Graecia* University of Catanzaro, 88100 Catanzaro, Italy; gcosta@unicz.it; 4 Department of Radiation Oncology, Cancer Biology Division, Washington University in St. Louis School of Medicine, St. Louis, MO 63108, USA; cinziafederico84@gmail.com (C.F.); kareem.azab@wustl.edu (A.K.A.); 5 IRCCS Istituto Ortopedico Rizzoli, 40136 Bologna, Italy; lavinia.raimondi@ior.it (L.R.); gianluca.giavaresi@ior.it (G.G.); 6 Interdepartmental Center of Services (CIS) of Genomics, Department of Experimental and Clinical Medicine, *Magna Graecia* University of Catanzaro, 88100 Catanzaro, Italy; 7 Department of Health Science, *Magna Graecia* University of Catanzaro, 88100 Catanzaro, Italy; 8 IRCCS, Ospedale Policlinico San Martino, 16100 Genoa, Italy; antonia.cagnetta@unige.it (A.C.); michele.cea@unige.it (M.C.); 9 Chair of Hematology, Department of Internal Medicine (DiMI), University of Genoa, 16100 Genoa, Italy; 10 Department of Oncology and Hemato-oncology, University of Milan and Hematology, Fondazione Cà Granda IRCCS Policlinico, 20122 Milan, Italy; antonino.neri@unimi.it

**Keywords:** multiple myeloma, non-coding RNA, plasma cell dyscrasia, miRNA, lncRNA

## Abstract

Despite substantial advancements have been done in the understanding of the pathogenesis of plasma cell (PC) disorders, these malignancies remain hard-to-treat. The discovery and subsequent characterization of non-coding transcripts, which include several members with diverse length and mode of action, has unraveled novel mechanisms of gene expression regulation often malfunctioning in cancer. Increasing evidence indicates that such non-coding molecules also feature in the pathobiology of PC dyscrasias, where they are endowed with strong therapeutic and/or prognostic potential. In this review, we aim to summarize the most relevant findings on the biological and clinical features of the non-coding RNA landscape of malignant PCs, with major focus on multiple myeloma. The most relevant classes of non-coding RNAs will be examined, along with the mechanisms accounting for their dysregulation and the recent strategies used for their targeting in PC dyscrasias. It is hoped these insights may lead to clinical applications of non-coding RNA molecules as biomarkers or therapeutic targets/agents in the near future.

## 1. Introduction

Plasma cell (PC) dyscrasias represent a clinically and biologically heterogeneous group of blood disorders characterized by the detection of a monoclonal paraprotein in the serum or urine, and/or the presence of monoclonal PCs in the bone marrow (BM) or in extramedullary tissues. This set of diseases include monoclonal gammopathy of undetermined significance (MGUS), multiple myeloma (MM), plasma cell leukemia (PCL), lymphoplasmacytic lymphoma/Waldenström macroglobulinemia (LPL/WM), amyloidosis and POEMS (Polyneuropathy, Organomegaly, Endocrinopathy, Monoclonal protein and Skin changes) syndrome.

MM is caused by the clonal proliferation of abnormal PCs in the BM, and represents around 10% of all hematological malignancies. Despite a vast improvement in treatment strategies over the last few decades, MM still remains incurable. Several risk factors associate with the disease, such as age, race, gender, family history [1] and obesity [2]. MM is always preceded by a premalignant condition known as MGUS, characterized by the finding of monoclonal protein or M-protein in serum (≤3 g/dL) and moderate PC proliferation (<10%) in the BM [3]. Despite M protein, MGUS patients lack “myeloma-defining events”, including CRAB symptoms (hypercalcemia, renal insufficiency, anemia and bone disease) [4]. MGUS prevalence is approximately 3% of the population aged over 50 years and increases with age [3]. It is estimated that around 1% of these patients will progress to MM each year. No treatment is indicated in MGUS patients, although a careful monitoring is required throughout life to early detect progression toward MM [5].

Four early oncogenic events have been described for MGUS and MM, that include translocations, dysregulation of cyclin D/retinoblastoma pathway, hyperdiploidy and chromosome 13 deletions. [6]. MGUS can progress to smoldering MM (sMM), which retains the diagnostic features of MM, but still lacks myeloma-defining events [4].

PCL is a distinct PC dyscrasia that can result as progression of symptomatic MM (secondary PCL) or as *de novo* disease (primary PCL), whose diagnosis relies on the presence of at least 2 × 10^9^/L or 20% circulating malignant PCs in the peripheral blood (PB). PCL accounts for around 0.5% cases of MM, with a crude incidence of 0.4 cases per million [7,8]. Treatments adopted resemble MM protocols, but overall outcomes of pPCLs and sPCLs are poorer [9,10].

WM is a B-cell malignancy classified as lymphoplasmacytic lymphoma [11], characterized by a clonal infiltration of lymphoplasmacytic cells within the BM and a serum IgM monoclonal component. A pre-malignant condition, defined as IgM MGUS, may precede a clinically active WM. It is characterized by less than 10% BM lymphoplasmacytic cells, less than 3g/dL of monoclonal IgM and a lack of clinical signs or symptoms secondary to the WM disease. Importantly, a rate of IgM MGUS-to-WM progression of about 2% per year has been reported [12]. In parallel, a smoldering WM (sWM) status may also exist, defining patients with BM lymphoplasmacytic infiltration of 10% or more, IgM monoclonal protein of 3 g/dL or more, in the absence of any sign or symptom of disease. Conversely, WM is characterized by the presence of more than 10% BM clonal lymphoplasmacytic cells, monoclonal IgM of any degree and end organ damage [3,12]. In rare cases, WM cells may also infiltrate the central nervous system, leading to the so-called Bing-Neel syndrome [13,14].

Amyloid light-chain (AL) amyloidosis refers to the extracellular tissue deposition of monoclonal light chain fibrils. Patients can have AL amyloidosis alone or in association with other PC disorders such as MGUS, MM and LPL/WM. The median age at presentation is 64 years with men accounting for 70% of the cases [15].

POEMS syndrome is a rare disorder affecting patients in the fifth to sixth decade of life, whose clinical manifestations are highly variable. According to the IMWG, the diagnosis of POEMS syndrome is made by the presence of two mandatory criteria: peripheral neuropathy clinically sensorimotor with evidence of axonal and demyelinating damage and monoclonal plasma cell disorder characterized by serum or urine monoclonal protein, often lambda restricted; BM biopsy might be unrevealing [16].

Diagnostic criteria used for the classification of PC malignancies, along with the most relevant therapeutic options, are briefly summarized in Table 1.

A complex genomic and epigenomic landscape characterizes PC dyscrasias, and recent findings underscore the pivotal contribution of non-coding RNAs (ncRNAs) to the malignant transformation [17]. Herein, we will provide an overview of the most relevant ncRNAs, of their mechanism of action and of their emerging biological and clinical impact within the PC dyscrasias scenario.

## 2. ncRNAs: Molecular Features

**Figure 1:**
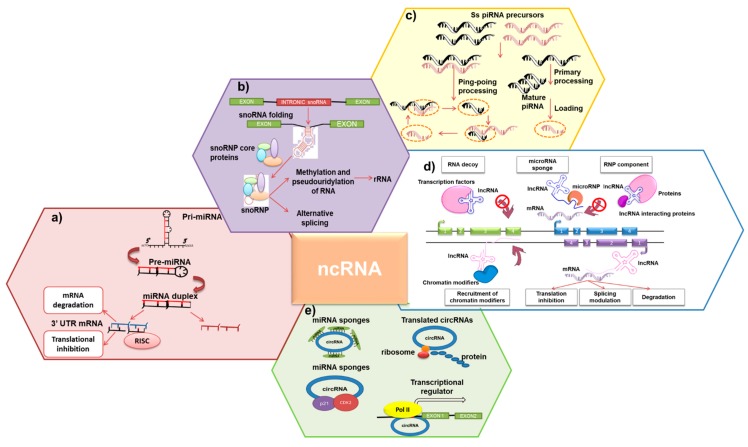
Molecular features and mechanism of action of the different ncRNA classes. (**a**) After being transcribed in the nucleus from a primary-miRNA (pri-miRNA), precursor miRNAs (pre-miRNAs) are exported by exportin 5 in the cytoplasm and processed by Dicer, which generates mature miRNAs, then loaded into the RNA-induced silencing complex (RISC). miRNAs function through degradation of protein-coding transcripts or translational repression. (**b**) Mature snoRNAs generated by splicing, debranching and trimming are either exported from the nucleus, where they regulate ribosomal RNA (rRNA) processing, or remain in the nucleus, where they can regulate alternative splicing. (**c**) piRNAs are expressed as single stranded RNAs (ss piRNAs) or produced through a secondary amplification loop. The PIWI ribonucleoprotein (piRNP) complex functions in transposon repression through target degradation and epigenetic silencing. (**d**) LncRNAs can modify gene expression by multiple mechanisms: they can act as decoy of transcription factors, sponge for miRNAs, regulators of splicing, recruiters of chromatin modifier complexes or modulate mRNA stability. (**e**) circRNAs can bind miRNAs acting as a sponge to regulate downstream transcription, or can enhance the expression of host genes by improving the activity of Pol II in the nucleus. Part of the circRNA can also encode peptides or proteins.

It is nowadays evident that the non-coding compartment, which represents approximately the 98.5% of the whole human transcriptome, critically regulates relevant physiologic and pathologic processes [18]. Based on their length, ncRNAs have been historically classified into short (<200 nucleotides) non-coding RNAs (sncRNAs) or long (>200 nucleotides) non-coding RNAs (lncRNAs) [19]. An overview of the mechanisms of action of each class is provided in Figure 1.

### 2.1. sncRNAs

(a) *miRNAs.* MicroRNAs (miRNAs) are sncRNA molecules, of 17 to 24 nucleotides (nt) in length, that post-transcriptionally regulate mRNAs [20,21]. After transcription by RNA polymerase II, miRNAs are processed through an evolutionarily conserved multi-step pathway, in which RNA endonucleases (Drosha and Dicer) progressively reduce the length of the initial miRNA transcripts from ~100 nt (primary-miRNA) to ~22 nt (mature miRNA). Mature miRNA is then assembled into the RNA-induced silencing complex (RISC) and can induce either translational repression or degradation of target mRNAs, upon total or partial complementary binding with 3′ untranslated region (3′ UTR) [21,22] (Figure 1a). Given the multitude of targets for a single miRNA, these molecules harbor the potential to concomitantly regulate multiple pathways [23]. As a consequence, dysregulation of miRNAs has been shown to underlie the onset and progression of cancers, including PC dyscrasias [24].

(b) *snoRNAs.* Small nucleolar RNAs (snoRNAs) are well conserved sncRNAs located in the nucleolus, commonly involved in post-transcriptional modification of ribosomal RNAs (rRNAs) and small nuclear RNA (snRNA) [25]. Interestingly, their regulatory activity goes far beyond the previous knowledge that they are transcriptionally and functionally related to the host genes within they are encoded. Indeed, “orphan” snoRNAs are also expressed in a tissue-specific fashion, and may be located within intron of lncRNAs or of coding genes unrelated to ribosomal biogenesis (Figure 1b) [26].

(c) *piRNAs.* PIWI-interacting RNAs (piRNAs) are sncRNAs formed by 25 to 31 nucleotides, that bind to the evolutionarily conserved proteins PIWIL1-4 [27]. In physiological conditions, both PIWILs and piRNAs are expressed in the germline, where they regulate transposable element and heterochromatin [28]. Unlike the other sncRNAs, piRNAs are generated from single-stranded RNA transcripts in a Dicer-independent mechanism. piRNAs have a preference for uridine at their 5′-ends, and have a HEN1-methyltransferase-catalyzed 2′-O-methylribose modification at their 3′-ends [29]. Three major classes of PIWI proteins have been implicated in a “ping-pong” amplification process, which creates antisense piRNAs that repress the transcript of origin [30,31]. Several studies revealed that mRNAs are also targeted by piRNAs at 3′UTR, leading to their degradation [32] (Figure 1c). A role for the PIWIL–piRNA complex has recently emerged in somatic cells, where their expression can be reactivated under pathological stimuli [33,34].

### 2.2. LncRNAs

LncRNAs comprise a heterogenous class of intergenic transcripts (lincRNAs), enhancer RNAs (eRNAs), and sense or antisense transcripts that overlap other genes [35]. LncRNAs have been proposed to exert diverse functions, including in *cis* or in *trans* transcriptional regulation, organization of nuclear domains and regulation of proteins or RNA molecules [35,36]. LncRNAs exert their functions by interacting with DNA, RNA or proteins [37]. The myriad functions that derive from such interactions are commonly categorized in four non-mutually exclusive archetypes: signals, decoy, guide, scaffold [37] (Figure 1d).

(a) *Signals.* Transcription of these lncRNAs occurs at a very specific time and place, to interpret cellular context and respond to diverse stimuli. Relevant examples of lncRNAs acting as signals are those implicated in genomic imprinting such as XIST, KCNQ1ot1 and Air [38,39]; or those activated in response to specific stimuli like DNA damage (LINC-p21, PANDA and NORAD) [40,41,42] or temperature decrease (COLDAIR and COOLAIR) [37,43,44].

(b) *Decoy*. These lncRNAs negatively regulate effector molecules including proteins or miRNAs [37]; they behave as “molecular sink” because their major function is to bind and titrate away the effector molecules, which in turn cannot exert their molecular functions. [37]. A compelling example of this class is MALAT1 (metastasis-associated lung adenocarcinoma transcript 1), which binds to and sequesters several serine/arginine (SR) splicing factors into nuclear speckles [45].

(c) *Guide*. These lncRNAs direct ribonucleoprotein (RNP) complexes to target genes *in cis* (neighboring genes) or *in trans* (distantly located genes) [37]. The RNP complex brought on by the lncRNAs include both repressive (e.g., polycomb) and activating (e.g., MLL) complexes, as well as transcription factors (e.g., TFIIB). Key examples of *cis*-acting lncRNAs are XIST, AIR, COLDAIR, CCND1 and HOTTIP; while HOTAIR, LincRNA-p21 and JPX are well characterized trans-acting lncRNAs [37].

(d) *Scaffold*. These lncRNAs serve as platforms upon which relevant molecular components are assembled. To carry out this function, lncRNAs belonging to this archetype should possess different domains that concomitantly bind various effector molecules [37]. A fascinating example of lncRNA scaffold is provided by TERC (telomerase RNA component), an essential component of telomerase, a specialized reverse transcriptase that plays a fundamental role in the maintenance of genome stability. In order to work properly, telomerase catalytic activity indeed requires the association of two universal subunits, namely the lncRNA TERC, that provides the template for repeat synthesis, and the catalytic protein subunit TERT (telomerase reverse transcriptase) [46].

### 2.3. CircRNAs

Circular RNAs (circRNAs) are highly conserved ncRNA species formed through a back-splicing mechanism, in which a 5′ splice donor attacks an upstream 3′ splice site, leading to a 3′-5′ phosphodiester bond [47,48,49]. The lack of free ends makes circRNAs more stable and resistant to exonucleases than linear RNAs [50,51,52].

Expression of circRNAs is finely regulated in time and space [50,53], with an exquisite disease-specific pattern [50,53]. Given the high stability of circRNAs in body fluids, several studies suggested their use as diagnostic and prognostic biomarkers [54]. Similar to other ncRNAs, it has been hypothesized for exosomal circRNAs a functional role in transmitting signals among cell populations of the tumor microenvironment [55,56]. Mechanistically, the presence of miRNA binding sites within their sequence allows circRNAs to function as miRNA decoys. However, the binding of a miRNA to a circRNA does not always correlate with the blockade of miRNA function, but can instead allow circRNAs to function as miRNAs reservoir [56]. Furthermore, circRNAs with RNA-binding protein motifs can act as protein decoys, or can facilitate the interaction between multiple proteins as scaffolds [57]. CircRNAs can bear AUG sites and internal ribosome entry sites elements, and some circRNAs can be translated in particular conditions [58,59]. Finally, some circRNAs are found located into the nucleus where they seem to be involved in transcription or in splicing events [60,61,62,63,64] (Figure 1E).

## 3. Therapeutic Targeting of ncRNAs in PC Dyscrasias

### 3.1. General Strategies for Targeting the ncRNAs

Different approaches may be used to therapeutically target the ncRNA network in cancer. Of note, ncRNA molecules may be either used as targets or drugs, depending on the role (tumor promoting or tumor suppressing) during malignant transformation. Strategies for targeting sncRNAs may widely differ from those used to target lncRNAs and specific approaches may be required to inhibit ncRNAs located in certain subcellular compartments (i.e., nuclei). Moreover, along with the already established and continuously evolving field of RNA therapeutics by antisense oligonucleotides (ASOs), the development of small molecule (SM) inhibitors of ncRNAs represent a new emerging field. Below, we summarize the most promising approaches developed so far.

*miRNA replacement (miRNAs as drugs).* The most useful tools adopted to enforce tumor suppressor miRNAs are small synthetic RNA duplexes mimicking endogenous miRNAs. Since the in vivo delivery of miRNA oligonucleotides as therapeutics is restricted because of charge density, molecular weight and degradation by nucleases [31], various viral and non-viral delivery systems have been developed, including liposomal formulations, which protect miRNAs from nucleases and are eventually decorated at the surface to confer tissue selectivity [31,65].

*miRNA inhibition (miRNAs as targets).* ASOs are small oligonucleotides capable to cross the cell membrane with RNA/DNA-based structures that selectively bind to RNA *via* Watson-Crick hybridization [66]. Chemical modifications have been applied to stabilize ASOs for in vivo delivery, such as the locked nucleic acid (LNA), bicyclic RNA analogues in which the furanose ring in the sugar backbone is locked into a RNA mimicking conformation through the introduction of a methylene bridge [67]. LNA inhibitors are endowed with nuclease resistance, increased binding affinity to the target and enhanced tissue uptake [31,67]. ASOs bearing complementary sequence to mature miRNAs have been used to stoichiometrically antagonize onco-miRNAs. This approach has the potential to effectively inhibit the activity of individual miRNAs (anti-miRs), as well as of different miRNAs belonging to the same family (seed-targeting tiny LNAs); or, further, to inhibit several unrelated miRNAs with oncogenic functions (miRNA sponges). Alternatively, as we recently proposed, the expression of clustered onco-miRNAs may also be antagonized using ASO-dependent RNase H-mediated degradation of miRNA primary transcript (i.e., LNA gapmeRs).

*RNA therapeutics for oncogenic lncRNAs.* RNA interference (RNAi) has been widely exploited to inhibit lncRNAs in cancer cells through RISC-mediated degradation. In order to achieve efficient in vivo knock-down with siRNAs, the above described chemical modifications that improve uptake, stability and binding affinity of ASOs have been adopted. In case the lncRNA sequence becomes unfavorable to siRNA targeting because of extensive secondary structure or nuclear localization, the use of ASOs is mandatory [68,69]. Advantages of ASOs over siRNAs include their independence on the RISC machinery, higher specificity and fewer off-target effects. Ribozymes or deozyribozymes, which bind to a complementary target sequence and catalyze the cleavage of the flanked RNA region, may alternatively be exploited for the targeting of lncRNAs [70,71].

*SM inhibitors of ncRNAs.* A new era in RNA targeting has started in late 1980s with the development of SMs, after the discovery that some drugs can bind the bacterial ribosomal RNA [72,73]. Regarding miRNA-targeting, the major goal has been the identification of SMs that specifically bind to miRNAs and/or to its precursors, thereby decreasing their levels. Gumireddy et al. first discovered a diazobenzene derivative that inhibits the transcription of the oncogenic pri-miR-21 [74]. Liu et al. then reported that the alkaloid sophocarpine antagonized Dicer-mediated processing of miR-21 [75]. Later on, a bleomycin A5 conjugate, which selectively inhibits Drosha processing of pri-miR-96, was discovered and found to upregulate the miR-96 pro-apoptotic target FOXO1, inducing breast cancer cell death [76]. Interestingly, Costales et al. identified a SM with overlapping affinity for the precursors of both miR-515 and miR-885, which share a common target motif; this compound was optimized to bind only miR-515 by Inforna, a computational approach that drives sequence-based design of SM targeting structured RNA [77], leading to the Targaprimir-515 molecule [78].

Regarding lncRNAs, although RNA three-dimensional (3D) structures provide different regions for the recognition and binding of SMs [79,80], the design is difficult because nucleic acids show a highly dynamic conformation and a repetitive surface, and their binding pockets are much more polar and exposed to solvents than proteins [81]. However, studies on viral RNA motifs indicated that ligands can bind discrete RNA pockets, pointing to the applicability of SMs to target RNAs [82,83,84,85]. Compared to ASOs, SMs offer superior pharmacokinetics (PK) and have the potential to specifically recognize RNA on the basis of their secondary or tertiary structure and independently of the sequence [86]. Overall, there are limited preclinical data on ncRNA targeting by SMs. Telomere repeat-containing RNA (TERRA) is a lncRNA transcribed from the sub-telomeric sequences, characterized by 5′-(UUAGGG)-3 repeats at its 3′ end, and involved in the maintenance and regulation of telomere’s homeostasis [87]. Its *r*(UUAGGG)_n_ sequence can fold into G-quadruplex (G4) conformation that is required for telomere heterochromatin formation in cancer cells [88]. Carboxypyridostatin, the first compound selectively targeting TERRA, was discovered through a template-directed in situ “click chemistry” approach [89,90,91,92].

MALAT1 has been defined as a promising anticancer target, because it is over-expressed and its knock-down produces strong anti-tumor effects in almost all tumor types [71]. A 1500 nt long segment at the 3′-end of MALAT1 was identified as a region responsible for its oncogenic activity [93]. The X-ray crystal structure of a 74 nt region at the 3′-end has been solved confirming a triple helix which confers stability to MALAT1 [94,95]. Such unique tertiary structure thus represents an excellent anti-cancer target for SMs. Accordingly, Donlic et al. synthesized an SM library based on the RNA binding scaffold diphenylfuran, which was used to selectively target MALAT1 triple helix [96]. Le Grice et al. also reported two new SMs targeting MALAT1, one of which unaffecting the triplex stability [97]. High-throughput screening also identified SMs disrupting the interaction of the lncRNA HOTAIR with its partner EZH2, leading to growth inhibition of breast and glioblastoma patient-derived xenografts [98,99].

**Figure 2:**
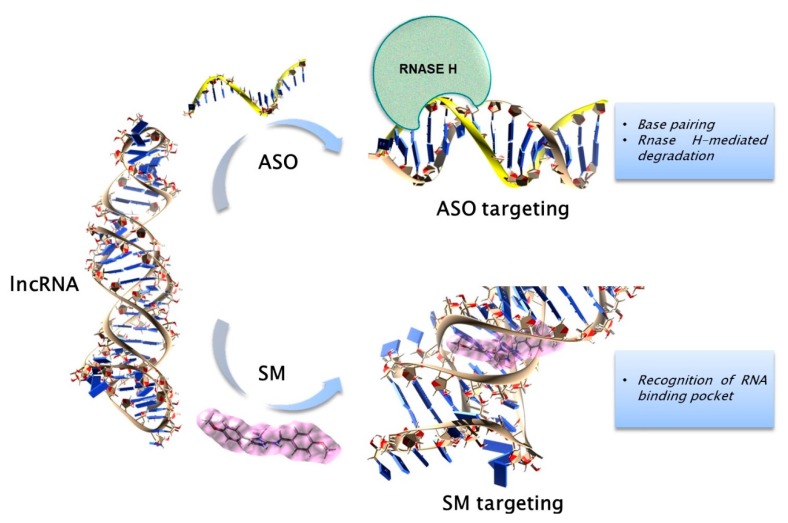
Strategies for lncRNA targeting. The picture reports, as a representative model, the triple helix region of the lncRNA MALAT1, which can be targeted or by LNA gapmeR ASO, that binds the lncRNA by perfect complementarity and triggers the RNAse H-mediated degradation (upper part), or by an SM designed to specifically recognize an RNA binding pocket (bottom part).

A cartoon reporting ASO- and SM-based lncRNA targeting approaches is reported in Figure 2.

### 3.2. Preclinical Findings on ncRNAs in PC Dyscrasis

#### 3.2.1. sncRNAs

During the last decade, extensive molecular profiling of tumor cells has shown deep dysregulation of miRNA networks in malignant PCs [100,101,102,103]. On this basis, the functional role of several miRNAs, either oncogenic or tumor suppressive, has been investigated in the preclinical setting, leading to a large body of evidence supporting their pivotal role in the pathogenesis of PC malignancies.

In MM, specific miRNA signatures characterize disease progression from MGUS to overt disease and eventually towards PCL [100,104]. For instance, oncogenic miR-21 and miR-106a-92 cluster were found upregulated starting from the MGUS stage, while other miRNAs were upregulated (e.g., miRs-221/222, -181a/b and -17-92 cluster) or downregulated (e.g., miR-15 and -16) predominantly in clinically manifest MM [104,105]. Among miRNAs upregulated in MM PCs, several reports demonstrated the oncogenic role of miR-221/222, a miRNA cluster acting *via* repression of CDKN1B (p27^Kip1^), BBC3 (PUMA), PTEN and CDKN1C (p57^Kip2^) [106,107,108]. Importantly, this cluster was shown to confer resistance to anti-MM agents including melphalan and dexamethasone [106,107,108]. Notably, the potential of miR-221 as therapeutic target was proved using the specific LNA-i-miR-221 inhibitor, that showed anti-MM efficacy and favorable PK profile upon systemic delivery in vivo [109]. On the other hand, enforced expression of tumor suppressor miR-34a exerted a strong anti-MM activity through BCL2, CDK6, and NOTCH1 targeting both in vitro an in vivo in the SCID-*synth-hu* model of MM, which recapitulates the disease within its BM *milieu* [110,111]. Likewise, the inverse correlation of miR-125b levels with IRF4, an “Achilles’ heel” of MM, showed that this specific miRNA, differently from other hematologic malignancies, has tumor suppressor activity in MM [112].

A specific miRNA signature markedly distinguished primary WM tumors from their normal counterparts, with a clear role for miR-155 as oncogenic driver and prognostic tool for this disease [102]. Functional studies showed that its inhibition by anti-miR-155 LNA decreased critical WM signaling cascades such as MAPK/ERK, PI3/AKT and NF-kB both in vitro and in vivo [113]. miRNA dysregulation, specifically involving miR-16, has also been correlated with AL amiloydosis, suggesting its oncogenic role, as well as its use as biomarker in the disease [103].

Genetic, epigenetic and transcriptional alterations are holding to be responsible for selective miRNA deregulation. Gene copy number alterations in both hyperdiploid MM (HMM) and non-HMM patients are one of the most prominent genomic perturbations [114], and miRNA expression is often altered upon gains and losses of chromosomal loci [115]. For instance, quantitative expression of miR-1232, miR-205, miR-215 and miR-488 strongly correlates with the 1q gain MM patients. miR-15a and -16 downregulation was similarly observed in patients displaying monosomy or deletions of chromosome 13, present in up to half of MM cases, with consequent induction of cell proliferation and BM angiogenesis [100,102,115].

Epigenetic modifications occurring during disease progression dynamically regulate the expression of miRNAs. In particular, DNA hypermethylation due to aberrant expression of *de novo* DNA methyltransferases (DNMTs) plays a pivotal role in MM pathogenesis [116]. Hypermethylation of promoters of a plethora of tumor suppressive miRNAs during MGUS to MM transition and toward PCL stage has been indeed reported [17]. Importantly, miR-155, found overexpressed in a variety of solid tumors [117], was hyper-methylated and down-regulated in MM cells [118], and its enforced expression by miR-155 mimics antagonized MM growth both in vitro and in vivo, and reduced proteasome activity through PSMβ5-targeting [119]. In addition, miR-29b, a tumor suppressor miRNA in hematological malignancies [120], was found silenced by EZH2-dependent H3K27 trimethylation [121] or by HDAC4-dependent deacetylation [122].

Besides being regulated by the epigenetic machinery, a subgroup of miRNAs, called “epi-miRNAs”, also actively regulates epigenetic processes via targeting mRNAs encoding methylating and acetylating enzymes [120]. DNMT3A/B are targeted by miRNAs, such as miR-29b, whose restoration efficiently reduces global DNA methylation leading to a strong anti-MM effect in vitro and in vivo in the SCID-*synth-hu* model [116], either as a single agent or in combination with demethylating agents [116], HDAC inhibitors [122] or proteasome inhibitors [123]. Of note, miR-29b also binds the 3′ UTR of the histone deacetylase HDAC4, thereby affecting the acetylation pattern of MM cells [122]. Similarly, a miRNA-dependent modulation of histone acetylation was described in WM, mainly due to aberrant expression of miRNA-206 and miR-9* in the tumor clone [124].

Expression of miRNAs is subjected to a tight regulation by numerous transcription factors [125]. Several transcription factor/miRNA autoregulatory loops have been described in MM, such as that between p53 and miR-194 or miR-34 [110,126], as well as the loop existing among transcription factor Sp1 and miR-29b which modulates bortezomib sensitivity [123]. Moreover, overexpression of c-MYC, a key driver of MM, appears to cause a widespread reorganization of miRNA expression patterns [127]. Importantly, c-MYC acts in concert with Sp1 to down-regulate the expression of tumor suppressor miR-23b in MM and WM cells [128], and miR-23b enforcement dampened *in vitro* and in vivo MM or WM growth. Additionally, c-MYC strongly induces the expression of miR-17-92 oncogenic cluster, which in turn regulates the expression of MYC target genes—including BCL2L11 (BIM)—establishing a homeostatic feed-forward loop (FFL) [129].

The interaction between miRNAs and transcription factors may be susceptible of pharmacologic intervention: a specific LNA gapmeR inhibitor that selectively targets MIR17HG primary transcript, named MIR17PTi, is able to disrupt the MYC/miR-17-92 FFL and to trigger apoptosis by inducing MYC-dependent synthetic lethality in patient-derived MM cells [129].

The MM microenvironment is composed by several immune cell types, with myeloid-derived suppressor cells (MDSCs) participating in immune suppression [130]. The pivotal role of MDSCs in MM was evidenced by their accumulation and activation in MM patients, as well as by their capacity to suppress T cells [131]. They have been also identified as pre-osteoclast cells and potential factor promoting MMBD and angiogenesis [132]. In MM, granulocytic-MDSCs significantly increased the stem-like cell proportion, sphere formation, and expression of stemness-related genes through a piRNA-823-dependent DNMT3A/B activation. Such stem traits were abrogated by a selective antagomiR-823 [133].

Effects of miR-based therapy have been investigated in the context of the BM microenvironment [134] and bone disease (BD) [135]. Specifically, replacement of miR-29b in osteoclasts prevented bone resorption by reducing expression of c-FOS and MMP2 [136]. Likewise, overexpression of miR-21 in BMSCs enhanced *in vitro* osteoclastogenesis, while its inhibition restored RANK-L/OPG balance via upregulation of its targets OPG and PIAS3, thus impairing osteoclast activity and reducing bone resorption [137]. Interestingly, miR-21 is also overexpressed in MM cells where it exerts a key oncogenic role downregulating PTEN and activating the AKT pathway. Its inhibition exerts strong anti-MM activity in vitro and in vivo [138]. miR-15a and -16-1 upregulation reduces MM cell adhesion to the stroma as well as migration in vitro [102]. Similarly, enforced expression of miR-29b reduces migration of both MM and endothelial cells *via* VEGF and IL-8 targeting [139]. Targeting of HIF-1α, a transcription factor overexpressed in the hypoxic MM microenvironment, *via* miR-199a-5p mimics, significantly impaired endothelial cells migration and MM-related angiogenesis [140]. Likewise, exosomal miR-135b promoted angiogenesis in MM *via* downregulation of factor-inhibiting HIF-1, and its targeting may represent an additional strategy to block angiogenesis [141].

The miRNA network is also dysregulated in MM-associated dendritic cells (DCs) and contributes to their tumor-promoting activity. Enforced expression miR-29b in DCs downregulated IL-23 in PCs also in the context of the SCID-*synth-hu* model, and antagonized the Th17 inflammatory response, thus restoring an efficient anti-MM immune microenvironment [142]. Preclinical data on other classes of ncRNAs in PC dyscrasias are quite scarce. As for miRNAs, a snoRNA fingerprint characterizes distinct molecular subtypes of MM as compared to the normal counterpart: a global snoRNA downregulation occurs from normal to MM and to PCL stage, with the TC2 group displaying overexpression of SNORD115 and SNORD116 families [143]. ACA11, a relevant oncogenic snoRNA in MM encoded within the intron of the WHSC1 gene overexpressed in t(4;14) MM [25], was found to be part of a snRNP complex which includes proteins involved in post-splicing intron complexes. ACA11 overexpression promoted MM cell proliferation, decreased oxidative stress and reinforced chemotherapy resistance through inhibition of NRF2, a transcriptional regulator of antioxidant response. The further characterization of snoRNAs in MM as well as in the other PC dyscrasias will shed light into additional oncogenic mechanisms driving these diseases [25,144].

Regarding piRNAs, piRNA-823 is the first investigated piRNA found overexpressed in MM patient-derived PCs, where high levels correlated with worse prognosis. Mechanistically, piRNA-823 affected the MM methylation profile, as demonstrated by inhibition of DNMTs in antagomiR-823 treated cells, which led to the reactivation of the hypermethylated tumor suppressor gene CDKN2A (p16^INK4A^). Of potential translational significance, antagomiR-823 delivery hampered cell growth in vitro triggering G0/G1 cell cycle arrest, apoptosis and reduced angiogenesis, as well as in vivo in relevant MM xenograft models [145]. MM-derived extracellular vesicles (EVs) containing piRNA-823 were found in the PB of MM patients and correlated with poor prognosis; such EVs promoted angiogenesis and invasion by transferring piRNA-823 to endothelial cells, as demonstrated in vitro and in vivo using xenograft models with endothelial cells treated either with antagomiR-823 or with piRNA-823-depleted EVs [146].

#### 3.2.2. LncRNAs

LncRNAs drive tumorigenesis by promoting all aspects or “hallmarks” of malignant transformation [147]. Their role in promoting the outset and progression of PC dyscrasias, however, is poorly defined. Major evidence has been provided only in MM, where three distinct transcriptomic analyses described a dysregulated lncRNA landscape. Ronchetti et al. used microarray technology to analyze lncRNA expression in patients at different stages of MM progression—including MGUS (20), sMM (33), MM (170) and PCL (36)—and in healthy donors (9) [148]. This study identified 31 lncRNAs altered in tumor samples compared to normal controls. Interestingly they found 21 lncRNAs, whose expression was deregulated proceeding toward the more aggressive stages of PC dyscrasias (PCL), suggesting a possible role in the disease progression [148]. In a follow up study, RNA-seq was used to evaluate lncRNA expression in 30 MM patients leading to the identification of 391 dysregulated lncRNAs [149]. The authors also provided a comprehensive catalogue of lncRNAs specifically associated with the main MM molecular subgroups and genetic alterations [149]. Samur et al. described the lncRNA landscape in MM cells by RNA-seq on MM PCs from 308 newly-diagnosed and uniformly treated patients enrolled to DFCI/IFM 2009 clinical study and on normal PCs from 16 HDs [150]. By comparing the expression of 7277 lncRNAs in these groups, they found 869 differentially expressed lncRNAs in MM compared to normal PCs. Among these, 395 were downregulated and 474 upregulated in MM cells [150]. They further observed a significant impact of copy number changes on lncRNA expression and identified a small subset of 14 lncRNAs strongly associated with progression free survival (PFS) [150]. The authors also developed a robust prognostic model to stratify patient risk, able to identify patients with differential outcomes within each low and high-risk categories using standard risk categorization, such as cytogenetic/FISH, ISS and MRD [150].

To date, only a few lncRNAs have been functionally investigated in MM—including MALAT1 [151,152,153], NEAT1 [154,155], CCAT1 [156] and H19 [157,158]. Importantly, different reports converge in defining the oncogenic role of MALAT1 in MM, which was found upregulated during the progression from intra-medullary to extra-medullary disease, with the higher levels associated with shorter OS and PFS [153]. We explored its functional role in MM and demonstrated that it may promote cell survival by regulating the expression and activity of the proteasome machinery [152]. Mechanistically, we established that MALAT1 interacts with EZH2 to regulate KEAP1 expression, triggering a KEAP1-dependent induction of NRF1/2, two relevant transcriptional activators of proteasome subunit genes [152]. As a corollary of our work, we provided evidence of MALAT1 druggability using LNA gapmeRs in vitro and in vivo in NOD-SCID mice bearing MM xenografts [152]. Hu et al. reported that MALAT1 acts as a scaffold in the formation of PARP1/LIG3 complexes that recognize DSBs on DNA and activate the alternative non-homologous end joining (A-NHEJ) DNA repair in MM cells [151]. Moreover, MALAT1 inhibition by ASOs was proven to synergize both with PARP and proteasome inhibitors, and a nanoparticle-based approach significantly increased the in vivo delivery of MALAT1 inhibitors [151]. NEAT1 is another lncRNA that has been deeply investigated in MM. It was firstly described as upregulated in primary MM cells [155], where it plays a key role in maintenance of genomic stability and DNA repair [154]. Of translational relevance, LNA gapmeR-mediated inhibition of NEAT1 antagonized growth of MM cells in NOD SCID mice, and synergized with conventional and novel anti-MM drugs [154].

#### 3.2.3. CircRNAs

CircRNAs are poorly characterized ncRNAs acting within complex regulatory systems involved in cancer pathogenesis [159]. A main obstacle to a deeper understanding of these molecules is the lack of standardized procedures. For instance, most public RNA-seq data sets come from poly(A) mRNAs enriched samples, limiting the annotation to circRNAs carrying poly(A) tail [160].

In hematological cancers, circRNAs have been proposed as valuable prognostic and diagnostic markers [161]. However, the biological impact of circRNAs in PC dyscrasias is insufficiently investigated and remains often unclear. Below, we summarize the more relevant examples of a functional role of circRNAs in MM.

The *circ_0000190* was found downregulated in primary MM cells and MM cell lines. Lower expression correlated with poorer survival rates of MM patients suggesting that it may affect disease behavior. In vitro and in vivo studies supported the tumor suppressor role of circ_0000190 that seems to exert its function by sponging miR-767-5p, preventing the repression of its validated target MAPK4; the anti-tumor activity of circ_0000190 was confirmed in mouse models [162].

The analysis of BM samples from 105 MM patients and 36 healthy controls highlighted MM downregulation of circRNA SWI/SNF-related matrix-associated actin dependent regulator of chromatin subfamily A member 5 (Circ-SMARCA5). Interestingly, expression levels of this circRNA negatively correlated with β2-microglobulin and the ISS stage, and elevated circ-SMARCA5 expression was associated with complete response to therapy and a better PFS and OS. Similar to circ_0000190, enforced expression of circ-SMARCA5 reduced MM cell proliferation and induced apoptosis by sponging miR-767-5p [163].

RNA-seq profiling of mantle cell lymphoma and MM cell lines revealed the expression of a large number of circRNAs including ciRS-7 [164], circHIPK3 [165,166], circSMARCA5 [167] and circZKSCAN1 [168]. Importantly, it was also identified a circRNA derived from IKZF3 that was not listed in circBase [169]. These circRNAs were then quantified by the NanoString technology in cell lines and patient samples from malignant B cells, including MM, providing a unique map of circRNA expression in B cell malignancies [170]. Among putative oncogenic circRNAs, circ_0007841 was found highly expressed in MM patient PCs, predicted worse PFS and correlated with chromosomal aberrations as 1q21 gain, t(4:14) and mutations in ATR and IRF4 genes [171].

The “competitive endogenous RNA” (ceRNA) hypothesis suggests that RNA transcripts communicate to each other by miRNA response elements (MREs), affecting the stability/translation of target RNAs by competing in miRNA binding [172]. ceRNAs are implicated in cancer [173], and circRNAs could play as ceRNAs: for instance, the endogenous circUBAP2 and hsa­_circ_0001892 both competed to inhibit the activity of miR-143, blocking apoptosis of MM cells [173].

A list of the most relevant ncRNAs studied in PC dyscrasias is reported in Table 2.

## 4. Circulating ncRNAs in PC Dyscrasias

Circulating ncRNAs have been identified in various body fluids [182]. They are quantifiable at low amounts, possess high stability as well as long-term storage properties [183] and are protected from enzymatic degradation through the binding with proteins or lipoproteins or through the packaging into EVs like exosomes [184,185,186].

Circulating ncRNA signatures might provide valuable information which integrates established disease markers with clinical features, thus contributing to a better diagnosis and prognostic stratification of PC dyscrasias’ patients [187].

The expression levels of circulating miRNAs may differ between serum samples of healthy controls and patients with asymptomatic or overt disease [187]. Jones et al. first identified circulating miR-720, miR-1308 and miR-1246 differently expressed in PB serum from patients with MGUS, MM and healthy donors; the combination of miR-720 and miR-1308 could discriminate healthy controls from MGUS and MM, whereas miR-1246 and miR-1308 combination distinguished MGUS from MM patients [188]. Kubiczkova et al. instead reported circulating miR-34a and let-7e levels as discriminating MM patients from normal controls, with a sensitivity of 80.6% and a specificity of 86.7%, and MGUS from healthy subjects with a sensitivity of 91.1% and a specificity of 96.7% [189]. Similarly, the combination of miR-19a and miR-4254 allowed the distinction of MM patients from healthy donors [190].

Furthermore, many studies underlined the prognostic potential of single circulating miRNAs. Plasma levels of miR-92a were significantly down-regulated in newly diagnosed MM patients as compared with healthy controls; moreover, levels of miR-92a changed in concordance with stage of the disease and response to treatment [191]. The expression of serum miR-29a was higher in MM patients with a sensitivity of 88% and a specificity of 70% than normal counterpart; nevertheless, its expression did not differ among patients at various ISS and DS stages [192].

Circulating miRNAs could also serve as predictive markers of MM survival. In fact, down-regulation of let-7e and miR-744 correlated with shortened survival and worse time to progression of MM patients [189]; conversely, lower levels of miR-19a positively correlated with either shorter PFS or OS, and ISS stage. Additionally, MM patients with low levels of miR-19a had a better response and extended survival after bortezomib treatment [190]. High serum levels of miR-16 and miR-25 positively correlated with better OS in MM patients, whereas high miR-25 correlated with better PFS [193]. Increased miR-483-5p levels were found in plasma of MM patients and associated with either PFS or ISS staging [194]; conversely, lower levels of circulating miR-130a were found in extramedullary myeloma (EMM) patients as compared with newly diagnosed and relapsed patients, and could discriminate EMM from MM patients or healthy donors [195].

High levels of serum miR-214 and miR-135b were found in patients with MMBD, where they positively correlated with the severity of the bone lytic lesions and predicted worst PFS and OS [196]; accordingly, high expression of miR-214 was detected in exosomes and serum of osteoporotic than non-osteoporotic patients, thus suggesting its potential as biomarker for MMBD [197]. A pattern of five circulating miRNAs was significantly reduced in patients with relapsed/refractory MM and poor responders after treatment with lenalidomide plus low-dose dexamethasone [198]. A relative lower expression of miR-143, miR-144, miR-199 and miR-203 in both BM supernatant fluid and serum of MM patients was found as compared with healthy controls; in these patients, levels of the proteoglycan VCAN positively, whereas miRNAs negatively correlated with the severity of disease [199].

Manier et al. isolated exosomal miRNAs from the serum of 156 newly diagnosed MM patients uniformly treated with bortezomib and dexamethasone, followed by high dose melphalan and autologous hematopoietic stem-cell transplantation. Let-7b and miR-18a levels significantly correlated with poorer outcomes in terms of PFS and OS [200].

Regarding exosomal miRNAs, the levels of serum exosome-derived miR-20a-5p, miR-103a-3p and miR-4505 were significantly different among patients with MM, patients with SMM and healthy individuals, while there were differences in the levels of let-7c-5p, miR-185-5p and miR-4741 in patients with MM relative to those in SMM patients or healthy controls [201].

More recently, circulating exosomes have been profiled also from patients with WM at different stages (30 sWM, 44 WM samples and 10 healthy controls), leading to the identification of a pattern of four exosomal miRNAs correlating with the disease status. Of note, the expression of let-7d decreased, whereas that of miR-192-5p, miR-21-5p and miR-320b increased during disease progression [202].

Regarding other ncRNAs, differential expression of five lncRNAs (TUG1, LincRNA-p21, MALAT1, HOTAIR, and GAS5) was observed between MM patients (*n* = 62) and healthy subjects (*n* = 40); TUG1 was the only found upregulated in the plasma of MM patients, whereas down-regulation of all the others was detected [203]. Recently, upregulation of lncRNA H19 was detected in the serum of MM patients (*n* = 80) as well as in MM cell lines (*n* = 3) as compared to their normal counterparts; H19 positively associated with DS and the ISS stage, and increased in bortezomib resistant patients [204].

## 5. Conclusions

Altogether, research thus far performed indicates that the malignant PC-associated ncRNA repertoire offers novel candidate biomarkers or targets for therapeutic intervention. Notably, evidence of the activity of certain classes of ncRNAs, such as lncRNAs, in PC dyscrasias different from MM is still at its infancy or lacking, and further studies are needed to validate their therapeutic and prognostic significance in these malignancies. We strongly believe that the ongoing development of novel tools to study and/or target ncRNAs will increase knowledge on their precise role in cancer pathobiology, strengthening the biological rationale underlying future ncRNA-based diagnostics and therapeutics.

## Figures and Tables

**Table 1:** Clinical features and best therapeutic options of PC dyscrasias.

PC Disorder	Bone Marrow PCs or Lymphoplasmacytic Cells, %	MC Serum/24 h FLC Urine	CRAB Features (Y/N) *	Best Therapeutic Options (First Line)
Symptomatic Multiple Myeloma	>10% PCs	> 3g/dL 500 mg	Y	PIs/ImiDs +/− MoAbs ** based regimens
Smoldering Multiple Myeloma	>10%<60% PCs	>or <3 g/dL/ 500 mg	N	No therapy−strict follow up
Plasma Cell Leukemia	>20% circulating PC in peripheral blood	> or <3 g/dL 500 mg	Y	PIs/ImiDs based regimens
MGUS	<10% PCs	<3 g/dL 500 mg	N	No therapy-follow up
Primary Amyloidosis	<10% PCs	<3 g/dL 500 mg	N	PIs/ImiDs +/− MoAbs ** based regimens
Solitary Plasmacytoma	<10% PCs	<3 g/dL 500 mg	Y ***	Radiotherapy
Smoldering Waldenström Macroglobulinemia	Usually <30% LPCs	<3g/dL	N	No therapy−strict follow up
Waldenström Macroglobulinemia	Usually >30% LPCs	>3 g/dL	N	PI based regimens+anti CD20 monoclonal; BTK inhibitors if MYD88^mut^
POEMS	>10% (in the case of an underlying MM)	>3 g/ dL (in the case of an underlying MM)	Y (in the case of an underlying MM)	MM regimens (****)

Abbreviations: FLC: free light chain; MC: monoclonal component; * CRAB: presence of at least one sign among the following: malignant hypercalcemia, renal failure, anemia, osteolytic bone lesions; clonal PC infiltration >60%; abnormal free light chain ratio (involved /uninvolved chain)>100; ** regimens including a proteasome inhibitor (PI) and /or an immunomodulatory molecule (thalidomide or lenalidomide, Imids) with a chemotherapy agent (e.g., cyclophosphamide, melphalan) or a monoclonal antibody (e.g., daratumumab, elotuzumab); *** only osteolytic bone lesion is considered; **** therapy depends on the presence of an underlying MM; NA: not applicable.

**Table 2:** Functionally characterized ncRNAs in PC dyscrasias.

Name	Class	Disease	Role in Tumorigenesis	Mechanisms *sncRNAs→targets* *lncRNAs→pathways*	References
Let-7b	miRNA	MM	Tumor-suppressor	MYC	[174]
miR-15a/16-1	miRNA	MM	Tumor-suppressor	MAP3KIP3, BCL2, AKT3, RPS6, VEGFA, IL17A, CABIN1	[105,175,176]
miR-17-92	miRNA	MM	Tumor-promoting	BCL2l11, TP53, PTEN, CDKN1A, SOCS1	[104,129]
miR-21	miRNA	MM	Tumor-promoting	PTEN, PIAS3	[137,138]
miR-22	miRNA	MM	Tumor-suppressor	LIG3	[177]
miR-29b	miRNA	MM	Tumor-suppressor	MCL1, CDK6, SP1, DNMT3A DNMT3B, FOS, MMP2	[116,122,123,136,139,142]
miR-34a	miRNA	MM	Tumor-suppressor	BCL2, CDK6, NOTCH1	[110]
miR-125a	miRNA	MM	Tumor-promoting	TP53	[178]
miR-125b	miRNA	MM	Tumor-suppressor	IRF4, PRDM1	[112]
miR-155	miRNA	MM	Tumor-suppressor	PSMβ5	[119]
miR-155	miRNA	WM	Tumor-promoting	CEBPB, SMAD5, SOCS1, MAFB, SHANK2, SH3PXD2A	[113]
miR-181a/b	miRNA	MM	Tumor-suppressor	KAT2B	[104]
miR-194-2-192	miRNA	MM	Tumor-suppressor	MDM2, IGF1	[126]
miR-199a-5p	miRNA	MM	Tumor-suppressor	HIF1A, VEGFA, CXCL8, FGF	[140]
miR-203	miRNA	MM	Tumor-suppressor	CREB-1	[179]
miR-215-194-1	miRNA	MM	Tumor-suppressor	MDM2, IGF1R	[126]
miR-214	miRNA	MM	Tumor-suppressor	PSMD10, ASF1B	[180]
miR-215	miRNA	MM	Tumor-suppressor	RUNX1	[181]
miR-221/222	miRNA	MM	Tumor-promoting	CDKN1B, CDKN1C, BBC3, PTEN	[106,107,109]
piRNA-823	piRNA	MM	Tumor-promoting	DNMTA, DNMT3B	[133]
ACA11	snoRNA	MM	Tumor-promoting	DHX9, ILF3, NCL, ADAR, HNRNPU	[25]
MALAT1	lncRNA	MM	Tumor-promoting	Transcriptional regulation of proteasome machinery; Activation of A-NHEJ DNA repair	[151,152]
NEAT1	lncRNA	MM	Tumor-promoting	Activation of HR DNA repair.	[154]
H19	lncRNA	MM	Tumor-promoting	Activation of NF-kB pathway; ceRNA of miR-29b-3p resulting in positive regulation of MCL1;	[157,158]
CCAT1	lncRNA	MM	Tumor-promoting	ceRNA of miR-181a-5p resulting in positive regulation of HOXA	[156]
circ_00001190	circRNA	MM	Tumor-suppressor	ceRNA of miR-767-5p resulting in upregulation of MAPK4	[162]
circ_SMARCA5	circRNA	MM	Tumor-suppressor	ceRNA of miR-767-5p	[163]

## References

[1] Beason T.S., Chang S.H., Sanfilippo K.M., Luo S., Colditz G.A., Vij R., Tomasson M.H., Dipersio J.F., Stockerl-Goldstein K., Ganti A. (2013). Influence of body mass index on survival in veterans with multiple myeloma. Oncologist.

[2] Morris E.V., Edwards C.M. (2018). Adipokines, adiposity, and bone marrow adipocytes: Dangerous accomplices in multiple myeloma. J. Cell Physiol..

[3] Kyle R.A., Larson D.R., Therneau T.M., Dispenzieri A., Kumar S., Cerhan J.R., Rajkumar S.V. (2018). Long-Term Follow-up of Monoclonal Gammopathy of Undetermined Significance. N Engl. J. Med..

[4] (2018). Multiple myeloma: 2018 update on diagnosis, risk-stratification, and management. Am. J. Hematol..

[5] Wadhera R.K., Rajkumar S.V. (2010). Prevalence of monoclonal gammopathy of undetermined significance: A systematic review. Mayo Clin. Proc..

[6] Gámez B., Edwards C.M. (2018). Contributions of the Bone Microenvironment to Monoclonal Gammopathy of Undetermined Significance Pathogenesis. Curr. Osteoporos. Rep..

[7] Sant M., Allemani C., Tereanu C., De Angelis R., Capocaccia R., Visser O., Marcos-Gragera R., Maynadié M., Simonetti A., Lutz J.M. (2010). Incidence of hematologic malignancies in Europe by morphologic subtype: Results of the HAEMACARE project. Blood.

[8] Gundesen M.T., Lund T., Moeller H.E.H., Abildgaard N. (2019). Plasma Cell Leukemia: Definition, Presentation, and Treatment. Curr. Oncol. Rep..

[9] Cifola I., Lionetti M., Pinatel E., Todoerti K., Mangano E., Pietrelli A., Fabris S., Mosca L., Simeon V., Petrucci M.T. (2015). Whole-exome sequencing of primary plasma cell leukemia discloses heterogeneous mutational patterns. Oncotarget.

[10] Chiecchio L., Dagrada G.P., White H.E., Towsend M.R., Protheroe R.K., Cheung K.L., Stockley D.M., Orchard K.H., Cross N.C., Harrison C.J. (2009). Frequent upregulation of MYC in plasma cell leukemia. Genes Chromosomes Cancer.

[11] Swerdlow S.H., Campo E., Pileri S.A., Harris N.L., Stein H., Siebert R., Advani R., Ghielmini M., Salles G.A., Zelenetz A.D. (2016). The 2016 revision of the World Health Organization classification of lymphoid neoplasms. Blood.

[12] Rajkumar S.V., Dispenzieri A., Kyle R.A. (2006). Monoclonal gammopathy of undetermined significance, Waldenström macroglobulinemia, AL amyloidosis, and related plasma cell disorders: Diagnosis and treatment. Mayo Clin. Proc..

[13] Owen R.G., Treon S.P., Al-Katib A., Fonseca R., Greipp P.R., McMaster M.L., Morra E., Pangalis G.A., San Miguel J.F., Branagan A.R. (2003). Clinicopathological definition of Waldenstrom’s macroglobulinemia: Consensus panel recommendations from the Second International Workshop on Waldenstrom’s Macroglobulinemia. Semin Oncol..

[14] Fonseca R., Hayman S. (2007). Waldenström macroglobulinaemia. Br J. Haematol..

[15] Vaxman I., Dispenzieri A., Muchtar E., Gertz M. (2019). New developments in diagnosis, risk assessment and management in systemic amyloidosis. Blood Rev..

[16] Jaccard A. (2018). POEMS Syndrome: Therapeutic Options. Hematol. Oncol. Clin. North Am..

[17] Amodio N., D’Aquila P., Passarino G., Tassone P., Bellizzi D. (2017). Epigenetic modifications in multiple myeloma: Recent advances on the role of DNA and histone methylation. Expert Opin. Ther. Targets.

[18] Mercer T.R., Dinger M.E., Mattick J.S. (2009). Long non-coding RNAs: Insights into functions. Nat. Rev. Genet..

[19] Djebali S., Davis C.A., Merkel A., Dobin A., Lassmann T., Mortazavi A., Tanzer A., Lagarde J., Lin W., Schlesinger F. (2012). Landscape of transcription in human cells. Nature.

[20] Bartel D.P. (2004). MicroRNAs: Genomics, biogenesis, mechanism, and function. Cell.

[21] Slack F.J., Chinnaiyan A.M. (2019). The Role of Non-coding RNAs in Oncology. Cell.

[22] Drusco A., Croce C.M. (2017). MicroRNAs and Cancer: A Long Story for Short RNAs. Adv. Cancer Res..

[23] Rupaimoole R., Slack F.J. (2017). MicroRNA therapeutics: Towards a new era for the management of cancer and other diseases. Nat. Rev. Drug Discov..

[24] Van Roosbroeck K., Calin G.A. (2017). Cancer Hallmarks and MicroRNAs: The Therapeutic Connection. Adv. Cancer Res..

[25] Chu L., Su M.Y., Maggi L.B., Lu L., Mullins C., Crosby S., Huang G., Chng W.J., Vij R., Tomasson M.H. (2012). Multiple myeloma-associated chromosomal translocation activates orphan snoRNA ACA11 to suppress oxidative stress. J. Clin. Invest..

[26] Taulli R., Pandolfi P.P. (2012). “Snorkeling” for missing players in cancer. J. Clin. Invest..

[27] Hashim A., Rizzo F., Marchese G., Ravo M., Tarallo R., Nassa G., Giurato G., Santamaria G., Cordella A., Cantarella C. (2014). RNA sequencing identifies specific PIWI-interacting small non-coding RNA expression patterns in breast cancer. Oncotarget.

[28] Martinez V.D., Vucic E.A., Thu K.L., Hubaux R., Enfield K.S., Pikor L.A., Becker-Santos D.D., Brown C.J., Lam S., Lam W.L. (2015). Unique somatic and malignant expression patterns implicate PIWI-interacting RNAs in cancer-type specific biology. Sci. Rep..

[29] Horwich M.D., Li C., Matranga C., Vagin V., Farley G., Wang P., Zamore P.D. (2007). The Drosophila RNA methyltransferase, DmHen1, modifies germline piRNAs and single-stranded siRNAs in RISC. Curr. Biol..

[30] Carmell M.A., Girard A., van de Kant H.J., Bourc’his D., Bestor T.H., de Rooij D.G., Hannon G.J. (2007). MIWI2 is essential for spermatogenesis and repression of transposons in the mouse male germline. Dev. Cell.

[31] Amodio N., Di Martino M.T., Neri A., Tagliaferri P., Tassone P. (2013). Non-coding RNA: A novel opportunity for the personalized treatment of multiple myeloma. Expert Opin. Biol. Ther..

[32] Rouget C., Papin C., Boureux A., Meunier A.C., Franco B., Robine N., Lai E.C., Pelisson A., Simonelig M. (2010). Maternal mRNA deadenylation and decay by the piRNA pathway in the early Drosophila embryo. Nature.

[33] Sellitto A., Geles K., D’Agostino Y., Conte M., Alexandrova E., Rocco D., Nassa G., Giurato G., Tarallo R., Weisz A. (2019). Molecular and Functional Characterization of the Somatic PIWIL1/piRNA Pathway in Colorectal Cancer Cells. Cells.

[34] Liu W., Gao Q., Chen K., Xue X., Li M., Chen Q., Zhu G., Gao Y. (2014). Hiwi facilitates chemoresistance as a cancer stem cell marker in cervical cancer. Oncol. Rep..

[35] Kopp F., Mendell J.T. (2018). Functional Classification and Experimental Dissection of Long Noncoding RNAs. Cell.

[36] Rinn J.L., Chang H.Y. (2012). Genome regulation by long noncoding RNAs. Annu Rev. Biochem.

[37] Wang K.C., Chang H.Y. (2011). Molecular mechanisms of long noncoding RNAs. Mol. Cell.

[38] Mohammad F., Mondal T., Kanduri C. (2009). Epigenetics of imprinted long non-coding RNAs. Epigenetics.

[39] Pontier D.B., Gribnau J. (2011). Xist regulation and function explored. Hum. Genet..

[40] Huarte M., Guttman M., Feldser D., Garber M., Koziol M.J., Kenzelmann-Broz D., Khalil A.M., Zuk O., Amit I., Rabani M. (2010). A large intergenic noncoding RNA induced by p53 mediates global gene repression in the p53 response. Cell.

[41] Hung T., Wang Y., Lin M.F., Koegel A.K., Kotake Y., Grant G.D., Horlings H.M., Shah N., Umbricht C., Wang P. (2011). Extensive and coordinated transcription of noncoding RNAs within cell-cycle promoters. Nat. Genet..

[42] Munschauer M., Nguyen C.T., Sirokman K., Hartigan C.R., Hogstrom L., Engreitz J.M., Ulirsch J.C., Fulco C.P., Subramanian V., Chen J. (2018). The NORAD lncRNA assembles a topoisomerase complex critical for genome stability. Nature.

[43] Heo J.B., Sung S. (2011). Vernalization-mediated epigenetic silencing by a long intronic noncoding RNA. Science.

[44] Swiezewski S., Liu F., Magusin A., Dean C. (2009). Cold-induced silencing by long antisense transcripts of an Arabidopsis Polycomb target. Nature.

[45] Tripathi V., Ellis J.D., Shen Z., Song D.Y., Pan Q., Watt A.T., Freier S.M., Bennett C.F., Sharma A., Bubulya P.A. (2010). The nuclear-retained noncoding RNA MALAT1 regulates alternative splicing by modulating SR splicing factor phosphorylation. Mol. Cell.

[46] Collins K. (2008). Physiological assembly and activity of human telomerase complexes. Mech. Ageing Dev..

[47] Barrett S.P., Wang P.L., Salzman J. (2015). Circular RNA biogenesis can proceed through an exon-containing lariat precursor. Elife.

[48] Schindewolf C., Braun S., Domdey H. (1996). In vitro generation of a circular exon from a linear pre-mRNA transcript. Nucleic Acids Res..

[49] Starke S., Jost I., Rossbach O., Schneider T., Schreiner S., Hung L.H., Bindereif A. (2015). Exon circularization requires canonical splice signals. Cell Rep..

[50] Jeck W.R., Sorrentino J.A., Wang K., Slevin M.K., Burd C.E., Liu J., Marzluff W.F., Sharpless N.E. (2013). Circular RNAs are abundant, conserved, and associated with ALU repeats. RNA.

[51] Conn S.J., Pillman K.A., Toubia J., Conn V.M., Salmanidis M., Phillips C.A., Roslan S., Schreiber A.W., Gregory P.A., Goodall G.J. (2015). The RNA binding protein quaking regulates formation of circRNAs. Cell.

[52] Ivanov A., Memczak S., Wyler E., Torti F., Porath H.T., Orejuela M.R., Piechotta M., Levanon E.Y., Landthaler M., Dieterich C. (2015). Analysis of intron sequences reveals hallmarks of circular RNA biogenesis in animals. Cell Rep..

[53] Salzman J., Gawad C., Wang P.L., Lacayo N., Brown P.O. (2012). Circular RNAs are the predominant transcript isoform from hundreds of human genes in diverse cell types. PLoS ONE.

[54] Wang F., Nazarali A.J., Ji S. (2016). Circular RNAs as potential biomarkers for cancer diagnosis and therapy. Am. J. Cancer Res..

[55] Li Y., Zheng Q., Bao C., Li S., Guo W., Zhao J., Chen D., Gu J., He X., Huang S. (2015). Circular RNA is enriched and stable in exosomes: A promising biomarker for cancer diagnosis. Cell Res..

[56] Rupaimoole R., Calin G.A., Lopez-Berestein G., Sood A.K. (2016). miRNA Deregulation in Cancer Cells and the Tumor Microenvironment. Cancer Discov..

[57] Du W.W., Fang L., Yang W., Wu N., Awan F.M., Yang Z., Yang B.B. (2017). Induction of tumor apoptosis through a circular RNA enhancing Foxo3 activity. Cell Death Differ..

[58] Legnini I., Di Timoteo G., Rossi F., Morlando M., Briganti F., Sthandier O., Fatica A., Santini T., Andronache A., Wade M. (2017). Circ-ZNF609 Is a Circular RNA that Can Be Translated and Functions in Myogenesis. Mol. Cell.

[59] Yang Y., Fan X., Mao M., Song X., Wu P., Zhang Y., Jin Y., Chen L.L., Wang Y., Wong C.C. (2017). Extensive translation of circular RNAs driven by N. Cell Res..

[60] Li Z., Huang C., Bao C., Chen L., Lin M., Wang X., Zhong G., Yu B., Hu W., Dai L. (2015). Exon-intron circular RNAs regulate transcription in the nucleus. Nat. Struct. Mol. Biol..

[61] Holdt L.M., Kohlmaier A., Teupser D. (2018). Molecular roles and function of circular RNAs in eukaryotic cells. Cell Mol. Life Sci..

[62] Wang Y., Zhang J., Li J., Gui R., Nie X., Huang R. (2019). CircRNA_014511 affects the radiosensitivity of bone marrow mesenchymal stem cells by binding to miR-29b-2-5p. Bosn. J. Basic Med. Sci..

[63] Chen L.L. (2016). The biogenesis and emerging roles of circular RNAs. Nat. Rev. Mol. Cell Biol..

[64] Zang J., Lu D., Xu A. (2018). The interaction of circRNAs and RNA binding proteins: An important part of circRNA maintenance and function. J. Neurosci. Res..

[65] Trang P., Wiggins J.F., Daige C.L., Cho C., Omotola M., Brown D., Weidhaas J.B., Bader A.G., Slack F.J. (2011). Systemic delivery of tumor suppressor microRNA mimics using a neutral lipid emulsion inhibits lung tumors in mice. Mol. Ther..

[66] Chery J. (2016). RNA therapeutics: RNAi and antisense mechanisms and clinical applications. Postdoc. J..

[67] Petersen M., Wengel J. (2003). LNA: A versatile tool for therapeutics and genomics. Trends Biotechnol..

[68] Ling H., Fabbri M., Calin G.A. (2013). MicroRNAs and other non-coding RNAs as targets for anticancer drug development. Nat. Rev. Drug Discov..

[69] Geary R.S. (2009). Antisense oligonucleotide pharmacokinetics and metabolism. Expert Opin. Drug Metab. Toxicol..

[70] Pavco P.A., Bouhana K.S., Gallegos A.M., Agrawal A., Blanchard K.S., Grimm S.L., Jensen K.L., Andrews L.E., Wincott F.E., Pitot P.A. (2000). Antitumor and antimetastatic activity of ribozymes targeting the messenger RNA of vascular endothelial growth factor receptors. Clin. Cancer Res..

[71] Amodio N., Raimondi L., Juli G., Stamato M.A., Caracciolo D., Tagliaferri P., Tassone P. (2018). MALAT1: A druggable long non-coding RNA for targeted anti-cancer approaches. J. Hematol. Oncol..

[72] Brosnan C.A., Voinnet O. (2009). The long and the short of noncoding RNAs. Curr. Opin. Cell Biol..

[73] Zhao F., Zhao Q., Blount K.F., Han Q., Tor Y., Hermann T. (2005). Molecular recognition of RNA by neomycin and a restricted neomycin derivative. Angew. Chem. Int. Ed. Engl..

[74] Gumireddy K., Young D.D., Xiong X., Hogenesch J.B., Huang Q., Deiters A. (2008). Small-molecule inhibitors of microrna miR-21 function. Angew. Chem. Int. Ed. Engl..

[75] Liu W., Zhang B., Chen G., Wu W., Zhou L., Shi Y., Zeng Q., Li Y., Sun Y., Deng X. (2017). Targeting miR-21 with Sophocarpine Inhibits Tumor Progression and Reverses Epithelial-Mesenchymal Transition in Head and Neck Cancer. Mol. Ther..

[76] Li Y., Disney M.D. (2018). Precise Small Molecule Degradation of a Noncoding RNA Identifies Cellular Binding Sites and Modulates an Oncogenic Phenotype. ACS Chem. Biol..

[77] Disney M.D., Winkelsas A.M., Velagapudi S.P., Southern M., Fallahi M., Childs-Disney J.L. (2016). Inforna 2.0: A Platform for the Sequence-Based Design of Small Molecules Targeting Structured RNAs. ACS Chem. Biol..

[78] Costales M.G., Hoch D.G., Abegg D., Childs-Disney J.L., Velagapudi S.P., Adibekian A., Disney M.D. (2019). A Designed Small Molecule Inhibitor of a Non-Coding RNA Sensitizes HER2 Negative Cancers to Herceptin. J. Am. Chem. Soc..

[79] Kligun E., Mandel-Gutfreund Y. (2013). Conformational readout of RNA by small ligands. RNA Biol..

[80] Kondo J., Westhof E. (2010). Base pairs and pseudo pairs observed in RNA-ligand complexes. J. Mol. Recognit..

[81] Warner K.D., Hajdin C.E., Weeks K.M. (2018). Principles for targeting RNA with drug-like small molecules. Nat. Rev. Drug Discov..

[82] Deigan K.E., Ferré-D’Amaré A.R. (2011). Riboswitches: Discovery of drugs that target bacterial gene-regulatory RNAs. Acc. Chem. Res..

[83] Blount K.F., Breaker R.R. (2006). Riboswitches as antibacterial drug targets. Nat. Biotechnol..

[84] Mei H.Y., Cui M., Heldsinger A., Lemrow S.M., Loo J.A., Sannes-Lowery K.A., Sharmeen L., Czarnik A.W. (1998). Inhibitors of protein-RNA complexation that target the RNA: Specific recognition of human immunodeficiency virus type 1 TAR RNA by small organic molecules. Biochemistry.

[85] Fernandes J., Jayaraman B., Frankel A. (2012). The HIV-1 Rev. response element: An RNA scaffold that directs the cooperative assembly of a homo-oligomeric ribonucleoprotein complex. RNA Biol..

[86] Connelly C.M., Moon M.H., Schneekloth J.S. (2016). The Emerging Role of RNA as a Therapeutic Target for Small Molecules. Cell Chem. Biol..

[87] Azzalin C.M., Reichenbach P., Khoriauli L., Giulotto E., Lingner J. (2007). Telomeric repeat containing RNA and RNA surveillance factors at mammalian chromosome ends. Science.

[88] Collie G.W., Parkinson G.N., Neidle S., Rosu F., De Pauw E., Gabelica V. (2010). Electrospray mass spectrometry of telomeric RNA (TERRA) reveals the formation of stable multimeric G-quadruplex structures. J. Am. Chem. Soc..

[89] Di Antonio M., Biffi G., Mariani A., Raiber E.A., Rodriguez R., Balasubramanian S. (2012). Selective RNA versus DNA G-quadruplex targeting by in situ click chemistry. Angew. Chem. Int. Ed. Engl..

[90] Shirude P.S., Gillies E.R., Ladame S., Godde F., Shin-Ya K., Huc I., Balasubramanian S. (2007). Macrocyclic and helical oligoamides as a new class of G-quadruplex ligands. J. Am. Chem. Soc..

[91] Rocca R., Talarico C., Moraca F., Costa G., Romeo I., Ortuso F., Alcaro S., Artese A. (2017). Molecular recognition of a carboxy pyridostatin toward G-quadruplex structures: Why does it prefer RNA?. Chem. Biol. Drug Des..

[92] Rocca R., Moraca F., Costa G., Nadai M., Scalabrin M., Talarico C., Distinto S., Maccioni E., Ortuso F., Artese A. (2017). Identification of G-quadruplex DNA/RNA binders: Structure-based virtual screening and biophysical characterization. Biochim. Biophys Acta Gen. Subj..

[93] Xu C., Yang M., Tian J., Wang X., Li Z. (2011). MALAT-1: A long non-coding RNA and its important 3′ end functional motif in colorectal cancer metastasis. Int. J. Oncol..

[94] Brown J.A., Bulkley D., Wang J., Valenstein M.L., Yario T.A., Steitz T.A., Steitz J.A. (2014). Structural insights into the stabilization of MALAT1 noncoding RNA by a bipartite triple helix. Nat. Struct. Mol. Biol..

[95] Wilusz J.E., JnBaptiste C.K., Lu L.Y., Kuhn C.D., Joshua-Tor L., Sharp P.A. (2012). A triple helix stabilizes the 3’ ends of long noncoding RNAs that lack poly(A) tails. Genes Dev..

[96] Donlic A., Morgan B.S., Xu J.L., Liu A., Roble C., Hargrove A.E. (2019). Corrigendum: Discovery of Small Molecule Ligands for MALAT1 by Tuning an RNA-Binding Scaffold. Angew. Chem. Int. Ed. Engl..

[97] Abulwerdi F.A., Xu W., Ageeli A.A., Yonkunas M.J., Arun G., Nam H., Schneekloth J.S., Dayie T.K., Spector D., Baird N. (2019). Selective Small-Molecule Targeting of a Triple Helix Encoded by the Long Noncoding RNA, MALAT1. ACS Chem. Biol..

[98] Ren Y., Wang Y.F., Zhang J., Wang Q.X., Han L., Mei M., Kang C.S. (2019). Targeted design and identification of AC1NOD4Q to block activity of HOTAIR by abrogating the scaffold interaction with EZH2. Clin. Epigenetics.

[99] Li Y., Ren Y., Wang Y., Tan Y., Wang Q., Cai J., Zhou J., Yang C., Zhao K., Yi K. (2019). A Compound AC1Q3QWB Selectively Disrupts HOTAIR-Mediated Recruitment of PRC2 and Enhances Cancer Therapy of DZNep. Theranostics.

[100] Lionetti M., Biasiolo M., Agnelli L., Todoerti K., Mosca L., Fabris S., Sales G., Deliliers G.L., Bicciato S., Lombardi L. (2009). Identification of microRNA expression patterns and definition of a microRNA/mRNA regulatory network in distinct molecular groups of multiple myeloma. Blood.

[101] Lionetti M., Musto P., Di Martino M.T., Fabris S., Agnelli L., Todoerti K., Tuana G., Mosca L., Gallo Cantafio M.E., Grieco V. (2013). Biological and clinical relevance of miRNA expression signatures in primary plasma cell leukemia. Clin. Cancer Res..

[102] Roccaro A.M., Sacco A., Chen C., Runnels J., Leleu X., Azab F., Azab A.K., Jia X., Ngo H.T., Melhem M.R. (2009). microRNA expression in the biology, prognosis, and therapy of Waldenstrom macroglobulinemia. Blood.

[103] Weng L., Spencer B.H., SoohHoo P.T., Connors L.H., O’Hara C.J., Seldin D.C. (2011). Dysregulation of miRNAs in AL amyloidosis. Amyloid.

[104] Pichiorri F., Suh S.S., Ladetto M., Kuehl M., Palumbo T., Drandi D., Taccioli C., Zanesi N., Alder H., Hagan J.P. (2008). MicroRNAs regulate critical genes associated with multiple myeloma pathogenesis. Proc. Natl. Acad. Sci. USA.

[105] Roccaro A.M., Sacco A., Thompson B., Leleu X., Azab A.K., Azab F., Runnels J., Jia X., Ngo H.T., Melhem M.R. (2009). MicroRNAs 15a and 16 regulate tumor proliferation in multiple myeloma. Blood.

[106] Di Martino M.T., Gulla A., Cantafio M.E., Lionetti M., Leone E., Amodio N., Guzzi P.H., Foresta U., Conforti F., Cannataro M. (2013). In vitro and in vivo anti-tumor activity of miR-221/222 inhibitors in multiple myeloma. Oncotarget.

[107] Gulla A., Di Martino M.T., Gallo Cantafio M.E., Morelli E., Amodio N., Botta C., Pitari M.R., Lio S.G., Britti D., Stamato M.A. (2016). A 13 mer LNA-i-miR-221 Inhibitor Restores Drug Sensitivity in Melphalan-Refractory Multiple Myeloma Cells. Clin. Cancer Res..

[108] Xu J., Su Y., Xu A., Fan F., Mu S., Chen L., Chu Z., Zhang B., Huang H., Zhang J. (2019). miR-221/222-Mediated Inhibition of Autophagy Promotes Dexamethasone Resistance in Multiple Myeloma. Mol. Ther..

[109] Di Martino M.T., Gulla A., Gallo Cantafio M.E., Altomare E., Amodio N., Leone E., Morelli E., Lio S.G., Caracciolo D., Rossi M. (2014). In vitro and in vivo activity of a novel locked nucleic acid (LNA)-inhibitor-miR-221 against multiple myeloma cells. PLoS ONE.

[110] Di Martino M.T., Leone E., Amodio N., Foresta U., Lionetti M., Pitari M.R., Cantafio M.E., Gulla A., Conforti F., Morelli E. (2012). Synthetic miR-34a mimics as a novel therapeutic agent for multiple myeloma: In vitro and in vivo evidence. Clin. Cancer Res..

[111] Zarone M.R., Misso G., Grimaldi A., Zappavigna S., Russo M., Amler E., Di Martino M.T., Amodio N., Tagliaferri P., Tassone P. (2017). Evidence of novel miR-34a-based therapeutic approaches for multiple myeloma treatment. Sci. Rep..

[112] Morelli E., Leone E., Cantafio M.E., Di Martino M.T., Amodio N., Biamonte L., Gulla A., Foresta U., Pitari M.R., Botta C. (2015). Selective targeting of IRF4 by synthetic microRNA-125b-5p mimics induces anti-multiple myeloma activity in vitro and in vivo. Leukemia.

[113] Zhang Y., Roccaro A.M., Rombaoa C., Flores L., Obad S., Fernandes S.M., Sacco A., Liu Y., Ngo H., Quang P. (2012). LNA-mediated anti-miR-155 silencing in low-grade B-cell lymphomas. Blood.

[114] Aktas Samur A., Minvielle S., Shammas M., Fulciniti M., Magrangeas F., Richardson P.G., Moreau P., Attal M., Anderson K.C., Parmigiani G. (2019). Deciphering the chronology of copy number alterations in Multiple Myeloma. Blood Cancer J..

[115] Misiewicz-Krzeminska I., Krzeminski P., Corchete L.A., Quwaider D., Rojas E.A., Herrero A.B., Gutierrez N.C. (2019). Factors Regulating microRNA Expression and Function in Multiple Myeloma. Noncoding RNA.

[116] Amodio N., Leotta M., Bellizzi D., Di Martino M.T., D’Aquila P., Lionetti M., Fabiani F., Leone E., Gulla A.M., Passarino G. (2012). DNA-demethylating and anti-tumor activity of synthetic miR-29b mimics in multiple myeloma. Oncotarget.

[117] Tam W., Dahlberg J.E. (2006). miR-155/BIC as an oncogenic microRNA. Genes Chromosomes Cancer.

[118] Krzeminski P., Sarasquete M.E., Misiewicz-Krzeminska I., Corral R., Corchete L.A., Martin A.A., Garcia-Sanz R., San Miguel J.F., Gutierrez N.C. (2015). Insights into epigenetic regulation of microRNA-155 expression in multiple myeloma. Biochim. Biophys Acta.

[119] Amodio N., Gallo Cantafio M.E., Botta C., Agosti V., Federico C., Caracciolo D., Ronchetti D., Rossi M., Driessen C., Neri A. (2019). Replacement of miR-155 Elicits Tumor Suppressive Activity and Antagonizes Bortezomib Resistance in Multiple Myeloma. Cancers (Basel).

[120] Amodio N., Rossi M., Raimondi L., Pitari M.R., Botta C., Tagliaferri P., Tassone P. (2015). miR-29s: A family of epi-miRNAs with therapeutic implications in hematologic malignancies. Oncotarget.

[121] Stamato M.A., Juli G., Romeo E., Ronchetti D., Arbitrio M., Caracciolo D., Neri A., Tagliaferri P., Tassone P., Amodio N. (2017). Inhibition of EZH2 triggers the tumor suppressive miR-29b network in multiple myeloma. Oncotarget.

[122] Amodio N., Stamato M.A., Gulla A.M., Morelli E., Romeo E., Raimondi L., Pitari M.R., Ferrandino I., Misso G., Caraglia M. (2016). Therapeutic Targeting of miR-29b/HDAC4 Epigenetic Loop in Multiple Myeloma. Mol. Cancer Ther..

[123] Amodio N., Di Martino M.T., Foresta U., Leone E., Lionetti M., Leotta M., Gulla A.M., Pitari M.R., Conforti F., Rossi M. (2012). miR-29b sensitizes multiple myeloma cells to bortezomib-induced apoptosis through the activation of a feedback loop with the transcription factor Sp1. Cell Death Dis..

[124] Roccaro A.M., Sacco A., Jia X., Azab A.K., Maiso P., Ngo H.T., Azab F., Runnels J., Quang P., Ghobrial I.M. (2010). microRNA-dependent modulation of histone acetylation in Waldenstrom macroglobulinemia. Blood.

[125] Calura E., Bisognin A., Manzoni M., Todoerti K., Taiana E., Sales G., Morgan G.J., Tonon G., Amodio N., Tassone P. (2016). Disentangling the microRNA regulatory milieu in multiple myeloma: Integrative genomics analysis outlines mixed miRNA-TF circuits and pathway-derived networks modulated in t(4;14) patients. Oncotarget.

[126] Pichiorri F., Suh S.S., Rocci A., De Luca L., Taccioli C., Santhanam R., Zhou W., Benson D.M., Hofmainster C., Alder H. (2010). Downregulation of p53-inducible microRNAs 192, 194, and 215 impairs the p53/MDM2 autoregulatory loop in multiple myeloma development. Cancer Cell.

[127] Chang T.C., Yu D., Lee Y.S., Wentzel E.A., Arking D.E., West K.M., Dang C.V., Thomas-Tikhonenko A., Mendell J.T. (2008). Widespread microRNA repression by Myc contributes to tumorigenesis. Nat. Genet..

[128] Fulciniti M., Amodio N., Bandi R.L., Cagnetta A., Samur M.K., Acharya C., Prabhala R., D’Aquila P., Bellizzi D., Passarino G. (2016). miR-23b/SP1/c-myc forms a feed-forward loop supporting multiple myeloma cell growth. Blood Cancer J..

[129] Morelli E., Biamonte L., Federico C., Amodio N., Di Martino M.T., Gallo Cantafio M.E., Manzoni M., Scionti F., Samur M.K., Gulla A. (2018). Therapeutic vulnerability of multiple myeloma to MIR17PTi, a first-in-class inhibitor of pri-miR-17-92. Blood.

[130] Pyzer A.R., Cole L., Rosenblatt J., Avigan D.E. (2016). Myeloid-derived suppressor cells as effectors of immune suppression in cancer. Int. J. Cancer.

[131] Malek E., de Lima M., Letterio J.J., Kim B.G., Finke J.H., Driscoll J.J., Giralt S.A. (2016). Myeloid-derived suppressor cells: The green light for myeloma immune escape. Blood Rev..

[132] Zhuang J., Zhang J., Lwin S.T., Edwards J.R., Edwards C.M., Mundy G.R., Yang X. (2012). Osteoclasts in multiple myeloma are derived from Gr-1+CD11b+myeloid-derived suppressor cells. PLoS ONE.

[133] Ai L., Mu S., Sun C., Fan F., Yan H., Qin Y., Cui G., Wang Y., Guo T., Mei H. (2019). Myeloid-derived suppressor cells endow stem-like qualities to multiple myeloma cells by inducing piRNA-823 expression and DNMT3B activation. Mol. Cancer.

[134] Raimondi L., De Luca A., Morelli E., Giavaresi G., Tagliaferri P., Tassone P., Amodio N. (2016). MicroRNAs: Novel Crossroads between Myeloma Cells and the Bone Marrow Microenvironment. Biomed. Res. Int..

[135] Bellavia D., Salamanna F., Raimondi L., De Luca A., Carina V., Costa V., Alessandro R., Fini M., Giavaresi G. (2019). Deregulated miRNAs in osteoporosis: Effects in bone metastasis. Cell Mol. Life Sci..

[136] Rossi M., Pitari M.R., Amodio N., Di Martino M.T., Conforti F., Leone E., Botta C., Paolino F.M., Del Giudice T., Iuliano E. (2013). miR-29b negatively regulates human osteoclastic cell differentiation and function: Implications for the treatment of multiple myeloma-related bone disease. J. Cell Physiol..

[137] Pitari M.R., Rossi M., Amodio N., Botta C., Morelli E., Federico C., Gulla A., Caracciolo D., Di Martino M.T., Arbitrio M. (2015). Inhibition of miR-21 restores RANKL/OPG ratio in multiple myeloma-derived bone marrow stromal cells and impairs the resorbing activity of mature osteoclasts. Oncotarget.

[138] Leone E., Morelli E., Di Martino M.T., Amodio N., Foresta U., Gulla A., Rossi M., Neri A., Giordano A., Munshi N.C. (2013). Targeting miR-21 inhibits in vitro and in vivo multiple myeloma cell growth. Clin. Cancer Res..

[139] Amodio N., Bellizzi D., Leotta M., Raimondi L., Biamonte L., D’Aquila P., Di Martino M.T., Calimeri T., Rossi M., Lionetti M. (2013). miR-29b induces SOCS-1 expression by promoter demethylation and negatively regulates migration of multiple myeloma and endothelial cells. Cell Cycle.

[140] Raimondi L., Amodio N., Di Martino M.T., Altomare E., Leotta M., Caracciolo D., Gulla A., Neri A., Taverna S., D’Aquila P. (2014). Targeting of multiple myeloma-related angiogenesis by miR-199a-5p mimics: In vitro and in vivo anti-tumor activity. Oncotarget.

[141] Umezu T., Tadokoro H., Azuma K., Yoshizawa S., Ohyashiki K., Ohyashiki J.H. (2014). Exosomal miR-135b shed from hypoxic multiple myeloma cells enhances angiogenesis by targeting factor-inhibiting HIF-1. Blood.

[142] Botta C., Cuce M., Pitari M.R., Caracciolo D., Gulla A., Morelli E., Riillo C., Biamonte L., Gallo Cantafio M.E., Prabhala R. (2018). MiR-29b antagonizes the pro-inflammatory tumor-promoting activity of multiple myeloma-educated dendritic cells. Leukemia.

[143] Ronchetti D., Todoerti K., Tuana G., Agnelli L., Mosca L., Lionetti M., Fabris S., Colapietro P., Miozzo M., Ferrarini M. (2012). The expression pattern of small nucleolar and small Cajal body-specific RNAs characterizes distinct molecular subtypes of multiple myeloma. Blood Cancer J..

[144] Mahajan N., Wu H.J., Bennett R.L., Troche C., Licht J.D., Weber J.D., Maggi L.B., Tomasson M.H. (2017). Sabotaging of the oxidative stress response by an oncogenic noncoding RNA. FASEB J..

[145] Yan H., Wu Q.L., Sun C.Y., Ai L.S., Deng J., Zhang L., Chen L., Chu Z.B., Tang B., Wang K. (2015). piRNA-823 contributes to tumorigenesis by regulating de novo DNA methylation and angiogenesis in multiple myeloma. Leukemia.

[146] Li B., Hong J., Hong M., Wang Y., Yu T., Zang S., Wu Q. (2019). piRNA-823 delivered by multiple myeloma-derived extracellular vesicles promoted tumorigenesis through re-educating endothelial cells in the tumor environment. Oncogene.

[147] Huarte M. (2015). The emerging role of lncRNAs in cancer. Nat. Med..

[148] Ronchetti D., Agnelli L., Taiana E., Galletti S., Manzoni M., Todoerti K., Musto P., Strozzi F., Neri A. (2016). Distinct lncRNA transcriptional fingerprints characterize progressive stages of multiple myeloma. Oncotarget.

[149] Ronchetti D., Agnelli L., Pietrelli A., Todoerti K., Manzoni M., Taiana E., Neri A. (2018). A compendium of long non-coding RNAs transcriptional fingerprint in multiple myeloma. Sci. Rep..

[150] Samur M.K., Minvielle S., Gulla A., Fulciniti M., Cleynen A., Aktas Samur A., Szalat R., Shammas M., Magrangeas F., Tai Y.T. (2018). Long intergenic non-coding RNAs have an independent impact on survival in multiple myeloma. Leukemia.

[151] Hu Y., Lin J., Fang H., Fang J., Li C., Chen W., Liu S., Ondrejka S., Gong Z., Reu F. (2018). Targeting the MALAT1/PARP1/LIG3 complex induces DNA damage and apoptosis in multiple myeloma. Leukemia.

[152] Amodio N., Stamato M.A., Juli G., Morelli E., Fulciniti M., Manzoni M., Taiana E., Agnelli L., Cantafio M.E.G., Romeo E. (2018). Drugging the lncRNA MALAT1 via LNA gapmeR ASO inhibits gene expression of proteasome subunits and triggers anti-multiple myeloma activity. Leukemia.

[153] Handa H., Kuroda Y., Kimura K., Masuda Y., Hattori H., Alkebsi L., Matsumoto M., Kasamatsu T., Kobayashi N., Tahara K.I. (2017). Long non-coding RNA MALAT1 is an inducible stress response gene associated with extramedullary spread and poor prognosis of multiple myeloma. Br J. Haematol..

[154] Taiana E., Favasuli V., Ronchetti D., Todoerti K., Pelizzoni F., Manzoni M., Barbieri M., Fabris S., Silvestris I., Gallo Cantafio M.E. (2019). Long non-coding RNA NEAT1 targeting impairs the DNA repair machinery and triggers anti-tumor activity in multiple myeloma. Leukemia.

[155] Taiana E., Ronchetti D., Favasuli V., Todoerti K., Manzoni M., Amodio N., Tassone P., Agnelli L., Neri A. (2019). Long non-coding RNA NEAT1 shows high expression unrelated to molecular features and clinical outcome in multiple myeloma. Haematologica.

[156] Chen L., Hu N., Wang C., Zhao H., Gu Y. (2018). Long non-coding RNA CCAT1 promotes multiple myeloma progression by acting as a molecular sponge of miR-181a-5p to modulate HOXA1 expression. Cell Cycle.

[157] Sun Y., Pan J., Zhang N., Wei W., Yu S., Ai L. (2017). Knockdown of long non-coding RNA H19 inhibits multiple myeloma cell growth via NF-kappaB pathway. Sci. Rep..

[158] Pan Y., Zhang Y., Liu W., Huang Y., Shen X., Jing R., Pu J., Wang X., Ju S., Cong H. (2019). LncRNA H19 overexpression induces bortezomib resistance in multiple myeloma by targeting MCL-1 via miR-29b-3p. Cell Death Dis..

[159] Shang Q., Yang Z., Jia R., Ge S. (2019). The novel roles of circRNAs in human cancer. Mol. Cancer.

[160] Kristensen L.S., Hansen T.B., Venø M.T., Kjems J. (2018). Circular RNAs in cancer: Opportunities and challenges in the field. Oncogene.

[161] Ji T., Chen Q., Tao S., Shi Y., Chen Y., Shen L., Wang C., Yu L. (2019). The research progress of circular RNAs in hematological malignancies. Hematology.

[162] Feng Y., Zhang L., Wu J., Khadka B., Fang Z., Gu J., Tang B., Xiao R., Pan G., Liu J. (2019). CircRNA circ_0000190 inhibits the progression of multiple myeloma through modulating miR-767-5p/MAPK4 pathway. J. Exp Clin. Cancer Res..

[163] Liu H., Wu Y., Wang S., Jiang J., Zhang C., Jiang Y., Wang X., Hong L., Huang H. (2019). Circ-SMARCA5 suppresses progression of multiple myeloma by targeting miR-767-5p. BMC Cancer.

[164] Weng W., Wei Q., Toden S., Yoshida K., Nagasaka T., Fujiwara T., Cai S., Qin H., Ma Y., Goel A. (2017). Circular RNA ciRS-7-A Promising Prognostic Biomarker and a Potential Therapeutic Target in Colorectal Cancer. Clin. Cancer Res..

[165] Zheng Q., Bao C., Guo W., Li S., Chen J., Chen B., Luo Y., Lyu D., Li Y., Shi G. (2016). Circular RNA profiling reveals an abundant circHIPK3 that regulates cell growth by sponging multiple miRNAs. Nat. Commun..

[166] Okholm T.L.H., Nielsen M.M., Hamilton M.P., Christensen L.L., Vang S., Hedegaard J., Hansen T.B., Kjems J., Dyrskjøt L., Pedersen J.S. (2017). Circular RNA expression is abundant and correlated to aggressiveness in early-stage bladder cancer. NPJ. Genom. Med..

[167] Barbagallo D., Caponnetto A., Cirnigliaro M., Brex D., Barbagallo C., D’Angeli F., Morrone A., Caltabiano R., Barbagallo G.M., Ragusa M. (2018). CircSMARCA5 Inhibits Migration of Glioblastoma Multiforme Cells by Regulating a Molecular Axis Involving Splicing Factors SRSF1/SRSF3/PTB. Int. J. Mol. Sci..

[168] Yao Z., Luo J., Hu K., Lin J., Huang H., Wang Q., Zhang P., Xiong Z., He C., Huang Z. (2017). ZKSCAN1 gene and its related circular RNA (circZKSCAN1) both inhibit hepatocellular carcinoma cell growth, migration, and invasion but through different signaling pathways. Mol. Oncol..

[169] Glažar P., Papavasileiou P., Rajewsky N. (2014). circBase: A database for circular RNAs. RNA.

[170] Dahl M., Daugaard I., Andersen M.S., Hansen T.B., Grønbæk K., Kjems J., Kristensen L.S. (2018). Enzyme-free digital counting of endogenous circular RNA molecules in B-cell malignancies. Lab. Investig..

[171] Gao M., Li C., Xiao H., Dong H., Jiang S., Fu Y., Gong L. (2019). hsa_circ_0007841: A Novel Potential Biomarker and Drug Resistance for Multiple Myeloma. Front. Oncol..

[172] Salmena L., Poliseno L., Tay Y., Kats L., Pandolfi P.P. (2011). A ceRNA hypothesis: The Rosetta Stone of a hidden RNA language?. Cell.

[173] Zhong Y., Du Y., Yang X., Mo Y., Fan C., Xiong F., Ren D., Ye X., Li C., Wang Y. (2018). Circular RNAs function as ceRNAs to regulate and control human cancer progression. Mol. Cancer.

[174] Manier S., Powers J.T., Sacco A., Glavey S.V., Huynh D., Reagan M.R., Salem K.Z., Moschetta M., Shi J., Mishima Y. (2017). The LIN28B/let-7 axis is a novel therapeutic pathway in multiple myeloma. Leukemia.

[175] Li Y., Zhang B., Li W., Wang L., Yan Z., Li H., Yao Y., Yao R., Xu K., Li Z. (2016). MiR-15a/16 regulates the growth of myeloma cells, angiogenesis and antitumor immunity by inhibiting Bcl-2, VEGF-A and IL-17 expression in multiple myeloma. Leuk. Res..

[176] Zhang L., Zhou L., Shi M., Kuang Y., Fang L. (2018). Downregulation of miRNA-15a and miRNA-16 promote tumor proliferation in multiple myeloma by increasing CABIN1 expression. Oncol. Lett..

[177] Caracciolo D., Di Martino M.T., Amodio N., Morelli E., Montesano M., Botta C., Scionti F., Talarico D., Altomare E., Gallo Cantafio M.E. (2019). miR-22 suppresses DNA ligase III addiction in multiple myeloma. Leukemia.

[178] Leotta M., Biamonte L., Raimondi L., Ronchetti D., Di Martino M.T., Botta C., Leone E., Pitari M.R., Neri A., Giordano A. (2014). A p53-dependent tumor suppressor network is induced by selective miR-125a-5p inhibition in multiple myeloma cells. J. Cell Physiol..

[179] Wong K.Y., Liang R., So C.C., Jin D.Y., Costello J.F., Chim C.S. (2011). Epigenetic silencing of MIR203 in multiple myeloma. Br J. Haematol..

[180] Misiewicz-Krzeminska I., Sarasquete M.E., Quwaider D., Krzeminski P., Ticona F.V., Paino T., Delgado M., Aires A., Ocio E.M., Garcia-Sanz R. (2013). Restoration of microRNA-214 expression reduces growth of myeloma cells through positive regulation of P53 and inhibition of DNA replication. Haematologica.

[181] Liu S., Zhang Y., Huang C., Lin S. (2020). miR-215-5p is an anticancer gene in multiple myeloma by targeting RUNX1 and deactivating the PI3K/AKT/mTOR pathway. J. Cell Biochem..

[182] Cortez M.A., Bueso-Ramos C., Ferdin J., Lopez-Berestein G., Sood A.K., Calin G.A. (2011). MicroRNAs in body fluids--the mix of hormones and biomarkers. Nat. Rev. Clin. Oncol..

[183] Mitchell P.S., Parkin R.K., Kroh E.M., Fritz B.R., Wyman S.K., Pogosova-Agadjanyan E.L., Peterson A., Noteboom J., O’Briant K.C., Allen A. (2008). Circulating microRNAs as stable blood-based markers for cancer detection. Proc. Natl. Acad. Sci. USA.

[184] Arroyo J.D., Chevillet J.R., Kroh E.M., Ruf I.K., Pritchard C.C., Gibson D.F., Mitchell P.S., Bennett C.F., Pogosova-Agadjanyan E.L., Stirewalt D.L. (2011). Argonaute2 complexes carry a population of circulating microRNAs independent of vesicles in human plasma. Proc. Natl. Acad. Sci. USA.

[185] Wang K., Zhang S., Weber J., Baxter D., Galas D.J. (2010). Export of microRNAs and microRNA-protective protein by mammalian cells. Nucleic Acids Res..

[186] Vickers K.C., Palmisano B.T., Shoucri B.M., Shamburek R.D., Remaley A.T. (2011). MicroRNAs are transported in plasma and delivered to recipient cells by high-density lipoproteins. Nat. Cell Biol..

[187] Federico C., Sacco A., Belotti A., Ribolla R., Cancelli V., Giacomini A., Ronca R., Chiarini M., Imberti L., Marini M. (2019). Circulating microRNAs and Their Role in Multiple Myeloma. Noncoding RNA.

[188] Jones C.I., Zabolotskaya M.V., King A.J., Stewart H.J., Horne G.A., Chevassut T.J., Newbury S.F. (2012). Identification of circulating microRNAs as diagnostic biomarkers for use in multiple myeloma. Br J. Cancer.

[189] Kubiczkova L., Kryukov F., Slaby O., Dementyeva E., Jarkovsky J., Nekvindova J., Radova L., Greslikova H., Kuglik P., Vetesnikova E. (2014). Circulating serum microRNAs as novel diagnostic and prognostic biomarkers for multiple myeloma and monoclonal gammopathy of undetermined significance. Haematologica.

[190] Hao M., Zang M., Wendlandt E., Xu Y., An G., Gong D., Li F., Qi F., Zhang Y., Yang Y. (2015). Low serum miR-19a expression as a novel poor prognostic indicator in multiple myeloma. Int. J. Cancer.

[191] Yoshizawa S., Ohyashiki J.H., Ohyashiki M., Umezu T., Suzuki K., Inagaki A., Iida S., Ohyashiki K. (2012). Downregulated plasma miR-92a levels have clinical impact on multiple myeloma and related disorders. Blood Cancer J..

[192] Sevcikova S., Kubiczkova L., Sedlarikova L., Slaby O., Hajek R. (2013). Serum miR-29a as a marker of multiple myeloma. Leuk. Lymphoma.

[193] Rocci A., Hofmeister C.C., Geyer S., Stiff A., Gambella M., Cascione L., Guan J., Benson D.M., Efebera Y.A., Talabere T. (2014). Circulating miRNA markers show promise as new prognosticators for multiple myeloma. Leukemia.

[194] Qu X., Zhao M., Wu S., Yu W., Xu J., Li J., Chen L. (2014). Circulating microRNA 483-5p as a novel biomarker for diagnosis survival prediction in multiple myeloma. Med. Oncol..

[195] Besse L., Sedlarikova L., Kryukov F., Nekvindova J., Radova L., Slaby O., Kuglik P., Almasi M., Penka M., Krejci M. (2015). Circulating Serum MicroRNA-130a as a Novel Putative Marker of Extramedullary Myeloma. PLoS ONE.

[196] Hao M., Zang M., Zhao L., Deng S., Xu Y., Qi F., An G., Qin Y., Sui W., Li F. (2016). Serum high expression of miR-214 and miR-135b as novel predictor for myeloma bone disease development and prognosis. Oncotarget.

[197] Sun W., Zhao C., Li Y., Wang L., Nie G., Peng J., Wang A., Zhang P., Tian W., Li Q. (2016). Osteoclast-derived microRNA-containing exosomes selectively inhibit osteoblast activity. Cell Discov..

[198] Jung S.H., Lee S.E., Lee M., Kim S.H., Yim S.H., Kim T.W., Min C.K., Chung Y.J. (2017). Circulating microRNA expressions can predict the outcome of lenalidomide plus low-dose dexamethasone treatment in patients with refractory/relapsed multiple myeloma. Haematologica.

[199] Gupta N., Kumar R., Seth T., Garg B., Sati H.C., Sharma A. (2019). Clinical significance of circulatory microRNA-203 in serum as novel potential diagnostic marker for multiple myeloma. J. Cancer Res. Clin. Oncol..

[200] Manier S., Liu C.J., Avet-Loiseau H., Park J., Shi J., Campigotto F., Salem K.Z., Huynh D., Glavey S.V., Rivotto B. (2017). Prognostic role of circulating exosomal miRNAs in multiple myeloma. Blood.

[201] Zhang Z.Y., Li Y.C., Geng C.Y., Zhou H.X., Gao W., Chen W.M. (2019). Serum exosomal microRNAs as novel biomarkers for multiple myeloma. Hematol. Oncol..

[202] Bouyssou J.M., Liu C.J., Bustoros M., Sklavenitis-Pistofidis R., Aljawai Y., Manier S., Yosef A., Sacco A., Kokubun K., Tsukamoto S. (2018). Profiling of circulating exosomal miRNAs in patients with Waldenström Macroglobulinemia. PLoS ONE.

[203] Isin M., Ozgur E., Cetin G., Erten N., Aktan M., Gezer U., Dalay N. (2014). Investigation of circulating lncRNAs in B-cell neoplasms. Clin. Chim. Acta.

[204] Pan Y., Chen H., Shen X., Wang X., Ju S., Lu M., Cong H. (2018). Serum level of long noncoding RNA H19 as a diagnostic biomarker of multiple myeloma. Clin. Chim Acta.

